# Association of Tranexamic Acid Administration With Mortality and Thromboembolic Events in Patients With Traumatic Injury: A Systematic Review and Meta-analysis

**DOI:** 10.1001/jamanetworkopen.2022.0625

**Published:** 2022-03-01

**Authors:** Vivien Karl, Sophie Thorn, Tim Mathes, Simone Hess, Marc Maegele

**Affiliations:** 1 Institute for Research in Operative Medicine, Faculty of Health, Department of Medicine, Witten/Herdecke University, Cologne, Germany; 2 School of Public Health and Preventive Medicine, Monash University, Melbourne, Australia; 3 Department of Emergency Medicine, Alfred Health, Melbourne, Australia; 4 Institute for Medical Statistics, University Medical Centre, Göttingen, Germany; 5 Department of Traumatology, Orthopaedic Surgery and Sports Traumatology, Cologne-Merheim Medical Centre, Witten/Herdecke University, Campus Cologne-Merheim, Cologne, Germany

## Abstract

**Importance:**

Tranexamic acid is widely available and used off-label in patients with bleeding traumatic injury, although the literature does not consistently agree on its efficacy and safety.

**Objective:**

To examine the association of tranexamic acid administration with mortality and thromboembolic events compared with no treatment or with placebo in patients with traumatic injury in the literature.

**Data Sources:**

On March 23, 2021, PubMed, Embase, and the Cochrane Library were searched for eligible studies published between 1986 and 2021.

**Study Selection:**

Randomized clinical trials and observational studies investigating tranexamic acid administration compared with no treatment or placebo among patients with traumatic injury and traumatic brain injury who were 15 years or older were included. Included studies were published in English or German. The electronic search yielded 1546 records, of which 71 were considered for full-text screening. The selection process was performed independently by 2 reviewers.

**Data Extraction and Synthesis:**

The study followed the Cochrane Handbook for Systematic Reviews of Interventions and the Preferred Reporting Items for Systematic Reviews and Meta-Analyses (PRISMA) guidelines. Data were extracted by 2 independent reviewers and pooled using the inverse-variance random-effects model.

**Main Outcomes and Measures:**

Outcomes were formulated before data collection and included mortality at 24 hours and 28 and 30 days (1 month) as well as the incidence of thromboembolic events and the amount of blood products administered. Owing to missing data, overall mortality was added and the amount of blood products administered was discarded.

**Results:**

Thirty-one studies with a total of 43 473 patients were included in the systematic review. The meta-analysis demonstrated that administration of tranexamic acid was associated with a significant decrease in 1-month mortality compared with the control cohort (risk ratio, 0.83 [95% CI, 0.71-0.97]; *I*^2^ = 35%). The results of meta-analyses for 24-hour and overall mortality and thromboembolic events were heterogeneous and could not be pooled. Further investigations on clinical heterogeneity showed that populations with trauma and trial conditions differed markedly.

**Conclusions and Relevance:**

These findings suggest that tranexamic acid may be beneficial in various patient populations with trauma. However, reasonable concerns about potential thromboembolic events with tranexamic acid remain.

## Key Points

**Question:**

Is the administration of tranexamic acid associated with mortality and thromboembolic events in patients with traumatic injury?

**Findings:**

This systematic review and meta-analysis included 31 randomized clinical trials and observational studies with 43 473 patients, among whom tranexamic acid was associated with lower 1-month mortality compared with the control cohort. Data from tranexamic acid studies, especially thromboembolic events, are largely heterogeneous.

**Meaning:**

These findings suggest that tranexamic acid may decrease mortality in patients with traumatic injury, but the incidence of thromboembolic events remains unclear because trials and populations with traumatic injury are often not comparable and data vary largely.

## Teaser

This systematic review and meta-analysis evaluates the association of tranexamic acid administration with mortality and thromboembolic events among patients with traumatic injury.

## Introduction

Tranexamic acid is a widely available and low-cost medication to limit and manage hemorrhage. Tranexamic acid is indicated for menorrhagia and bleeding in patients with hemophilia^1^ but also commonly administered off-label for bleeding related to cardiac surgery, orthopedic surgery, and traumatic injuries.^1^ Many studies^2^ have sought to confirm or refute the hypothesis that tranexamic acid improves morbidity and mortality among the severely injured. In 2020, the results from 2 new randomized clinical trials (RCTs) on tranexamic acid in trauma^3,4^ were published. The present work was performed to provide an updated, high-quality systematic review and meta-analysis on the use of tranexamic acid in patients with trauma.

This systematic review and meta-analysis aimed to evaluate the association of tranexamic acid with mortality and the incidence of thromboembolic events among patients with traumatic injury by pooling data from RCTs and observational studies. Specific focus was given to the investigation of the heterogeneity in existing studies to provide insight into the complexity of results from meta-analyses that have yet to be conducted in the field of trauma research.

## Methods

This systematic review and meta-analysis followed the recommendations in the *Cochrane Handbook for Systematic Reviews of Interventions*^5^ and reported findings according to the Preferred Reporting Items for Systematic Reviews and Meta-analyses (PRISMA) reporting guideline. The study protocol has been registered in PROSPERO (CRD42021219835); all changes to the protocol are reported in the Methods section.

### Eligibility Criteria

Eligible patients were 15 years or older who presented to the emergency department with traumatic injuries and/or traumatic brain injury (TBI). The intervention investigated was treatment with intravenous tranexamic acid of any dosage regimen. The control group was treated with placebo or non-antifibrinolytic drugs. The primary outcome consisted of mortality at 24 hours and 28 and 30 days (1 month). The secondary outcomes were thromboembolic events and the amount of blood products given. Thromboembolic events included venous thromboembolism (eg, deep venous thrombosis), pulmonary embolism, and arterial thromboembolism (eg, myocardial infarction and stroke). Randomized clinical trials and observational studies with a control group were eligible. Furthermore, only trials published in English or German were included because these were common languages to the authors.

### Information Sources

On March 23, 2021, PubMed, Embase, and the Cochrane Library were searched for eligible studies published from 1986—when tranexamic acid was officially approved and licensed for medical use—to 2021. References of systematic reviews identified from the search were subsequently screened manually. Information on the search strategy is presented in the eMethods in the Supplement. We conducted post hoc analyses, including the additional outcome overall mortality, a subgroup analysis standard vs nonstandard administration, and a sensitivity analysis for RCTs vs observational studies.

### Selection Process

The selection process was performed independently by 2 reviewers (V.K and S.T.) and is summarized in eFigure 1 in the Supplement. Further information on the selection process is presented in the eMethods in the Supplement.

### Data Collection Process

Data were extracted by 1 reviewer (V.K.) and checked and verified by a second reviewer (S.T.). Disagreements were discussed until a consensus was reached. Information on the extracted data items is presented in the eMethods in the Supplement.

### Study Risk of Bias Assessment

A risk of bias assessment was conducted using the Cochrane Risk of Bias 2 tool for RCTs^5,6^ and the Critical Appraisal Skills Programme tool for observational studies.^7^ In the Cochrane Risk of Bias 2 tool, each domain can score low risk if there is no indication for risk of bias, some concerns if there is potential for risk of bias, or high risk if there is clear indication for risk of bias.^6^ Similarly, in the Critical Appraisal Skills Programme tool, each of the 12 domains can score low risk, some concerns, or high risk of bias.^7^ Two reviewers (V.K. and S.T.) applied the risk of bias assessment independently. Discrepancies were discussed until consensus.

### Reporting Bias Assessment

The Cochrane Risk of Bias 2 and the Critical Appraisal Skills Programme tools promoted the assessment of reporting bias. To assess reporting bias within studies, the list of outcomes reported in the protocols or Methods sections were compared with the outcomes reported in the published report. Publication bias across studies through visual inspection of funnel plots for asymmetry was assessed if at least 10 trials for each outcome were available.

### Statistical Analysis

We performed a meta-analysis using a random-effects model. We calculated the *P* value using the Egger test and pooled data only if the *P* value of the statistical test for heterogeneity was >.05. To describe statistical heterogeneity, we calculated *I*^2^ and 95% prediction intervals (PIs), and all results are presented with 95% CIs. We performed inverse variance random-effects meta-analyses using the Hartung-Knapp method and the Paule-Mandel heterogeneity variance estimator.^8,9^ For outcomes for which only sparse data were available (event rate <5%, no event studies, fewer than 5 RCTs in a meta-analysis), we used beta-binomial regression models for sensitivity analyses.^10,11^ We used the R software package meta, version 9.4, for the meta-analyses.^12^ Results are reported with 95% CIs, and no a priori threshold for statistical significance was established. The outcomes of mortality and thromboembolic events were dichotomous. We extracted raw data on events and the number of patients for each group and calculated relative risks if these were not available.

We conducted subgroup analyses to investigate differences between clinical heterogeneity and outcome as they pertained to the timing of tranexamic acid administration and injury characteristics. These analyses focused on patients in the following categories: (1) multiple trauma, including TBI patients vs predominantly TBI patients (described as multiple trauma patients vs TBI patients); (2) severely injured (ie, Injury Severity Score [ISS] ≥16, massive transfusion requirement, signs of shock) vs nonseverely injured patients; (3) blunt vs penetrating trauma; (4) in-hospital vs prehospital tranexamic acid administration; and (5) administration of tranexamic acid within 3 hours of injury vs beyond 3 hours of injury. For sensitivity analyses, the results from RCTs and observational studies were compared, excluding observational studies at high risk for confounding bias.

## Results

### Study Selection

The process of study selection is displayed in the PRISMA flow diagram (eFigure 1 in the Supplement). Further information and a list of excluded studies are provided in eResults and eTable 1 in the Supplement. Overall, 43 473 patients were included in this review, of whom 20 248 were treated with tranexamic acid and 23 225 without tranexamic acid.

### Study Characteristics

Of the 31 included studies, 6 were RCTs^2,3,4,13,14,15^ and 25 were observational studies.^16,17,18,19,20,21,22,23,24,25,26,27,28,29,30,31,32,33,34,35,36,37,38,39,40^ Information on study characteristics in terms of subgroups and countries in which the trials took place are provided in the eResults in the Supplement. A brief summary of the baseline characteristics of the study participants is given in Table 1. Detailed characteristics of each included study are summarized in eTable 2 in the Supplement.

**Table 1.** Baseline Characteristics

Source	Age, mean, y		Sex, % male		Country
	Intervention	Control	Intervention	Control	
Shakur et al,^2^ 2010	34.6	34.5	83.6	84.0	40 countries worldwide
Guyette et al,^3^ 2020	41	42	73.2	74.8	US
Rowell et al,^4^ 2020	39^a^	36^a^	73	75	US and Canada
CRASH-3 trial collaborators,^13^ 2019	41.7	41.9	80	80	29 countries worldwide
Chakroun-Walha et al,^14^ 2019	44	39	57	57	Tunisia
Yutthakasemsunt et al,^15^ 2013	34.8	34.1	86	91	Thailand
Bardes et al,^16^ 2017	NR	NR	NR	NR	US
Boutonnet et al,^17^ 2018	42	42	73.6	73.1	France
Chan et al,^18^ 2019	66.4	66.4	62.9	62.9	China
Cole et al,^19^ 2015	42	40	78	82	UK
Dixon et al,^20^ 2019	41	42	47	43	US
El-Menyar et al,^21^ 2020	31.4	31.5	96.1	89.2	Qatar
Glover et al,^22^ 2019	45.3	51.9	75.7	72.1	UK
Harvin et al,^23^ 2015	37	32	80	74	US
Howard et al,^24^ 2017	24.6	24.9	97.1	95.4	US
Johnston et al,^25^ 2018	25.3	27.4	99.3	96.4	US
Khan et al,^26^ 2018	42.5	38.7	66	68	US
Lewis et al,^27^ 2016	24.2	24.2	90.7	91.1	US
Luehr et al,^28^ 2017	41.6	41.9	42	36	US
Moore et al,^29^ 2017	27	34	85	77	US
Morrison et al,^30^ 2012	23.8	22.9	98.4	96.9	UK
Morrison et al,^31^ 2013	24.2	23.6	96.6	93.7	UK
Morte et al,^32^ 2019	24.7	25.3	100	100	US
Myers et al,^33^ 2019	36	32	75	70	US
Neeki et al,^34^ 2018	38	37.6	80.9	80.9	US
Neeki et al,^35^ 2020	38.9	37.9	84.3	86.1	US
Shiraishi et al,^36^ 2017	57^a^	56^a^	72.4	74.4	Japan
Swendsen et al,^37^ 2013	44.6	47.6	37	49	US
Valle et al,^38^ 2014	42	43	85	86	US
Wafaisade et al,^39^ 2016	43	41	72.5	72.5	Germany
Walker et al,^40^ 2020	24.2	25.5	NR	NR	US

Abbreviations: CRASH-3, Clinical Randomisation of an Antifibrinolytic in Significant Head Injury; NR, not reported.

^a^ Expressed as median.

### Outcomes

Owing to missing data, the outcome of overall mortality was added and the outcome of amount of blood products given was discarded. Changes and data are described in detail in the eResults and eTable 3 in the Supplement.

### Risk Assessment

**Figure 1.**
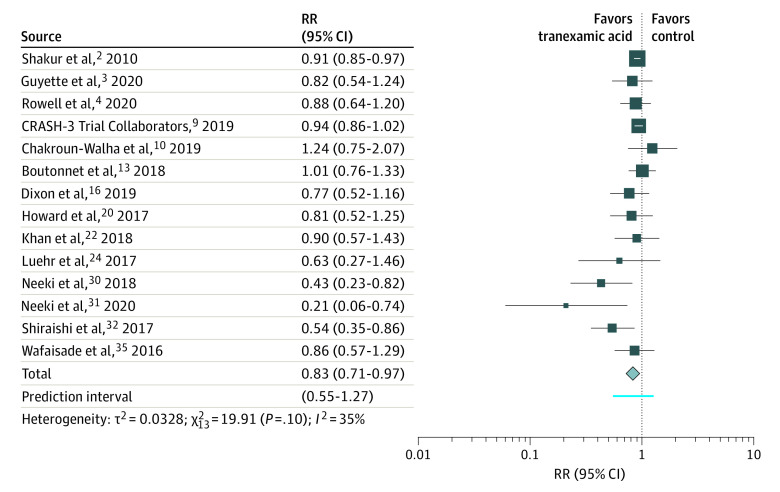
Forest Plot of 1-Month Mortality CRASH-3 indicates Clinical Randomisation of an Antifibrinolytic in Significant Head Injury; RR, rate ratio. Different size markers indicate weights used in meta-analyses and are proportional to study size.

**Figure 2.**
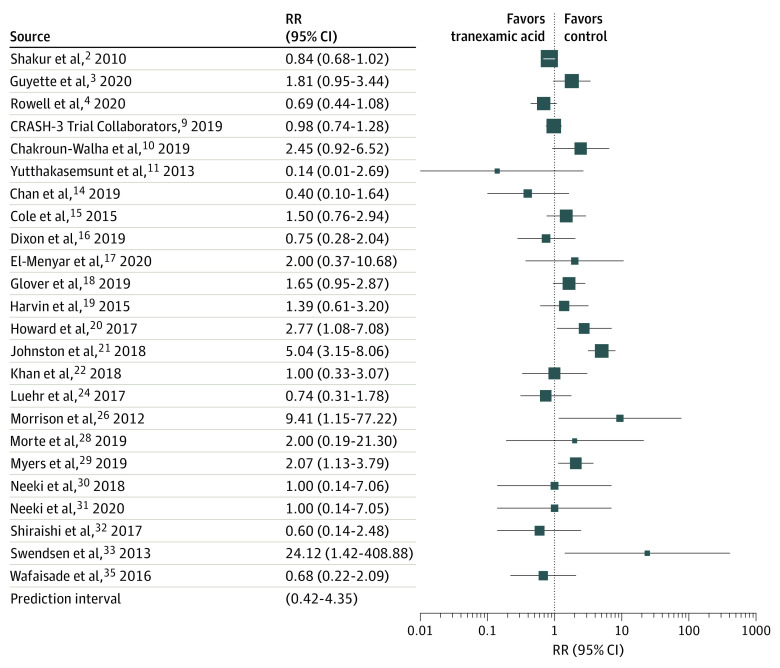
Forest Plot of Thromboembolic Events CRASH-3 indicates Clinical Randomisation of an Antifibrinolytic in Significant Head Injury; RR, rate ratio. Different size markers indicate weights used in meta-analyses and are proportional to study size.

The results of the risk of bias assessment for RCTs and observational studies are presented in Table 2 and Table 3, respectively. Two RCTs^2,13^ were rated as good quality with low risk, whereas there was some concern with 4 RCTs.^3,4,14,15^ Nine observational studies^22,23,25,27,28,29,30,37,40^ had a high risk of bias, mainly owing to inadequate handling of confounders, specifically relevant differences in baseline characteristics between intervention and control groups. All results of the meta-analysis are shown in the forest plots in Figure 1, Figure 2, and eFigures 2 to 27 in the Supplement.

**Table 2.** Risk of Bias Among Randomized Clinical Trials

Source	Risk of bias^a^					
	Randomization process	Deviations from intended interventions	Missing outcome data	Measurement of the outcome	Selection of the reported result	Overall
Shakur et al,^2^ 2010	Low	Low	Low	Low	Low	Low
Guyette et al,^3^ 2020	Low	Low	Low	Low	Some concerns	Some concerns
Rowell et al,^4^ 2020	Low	Low	Low	Low	Some concerns	Some concerns
CRASH-3 trial collaborators,^13^ 2019	Low	Low	Low	Low	Low	Low
Chakroun-Walha et al,^14^ 2019	Low	Some concerns	Low	Low	Some concerns	Some concerns
Yutthakasemsunt et al,^15^ 2013	Low	Low	Low	Low	Some concerns	Some concerns

Abbreviation: CRASH-3, Clinical Randomisation of an Antifibrinolytic in Significant Head Injury.

^a^ Assessed using the Cochrane Risk of Bias 2 tool.^5,6^

**Table 3.** Risk of Bias Among Observational Studies

Source	Study aspects scored for risk of bias^a^											
	Focus	Cohort selection	Exposure measurement	Outcome measurement	Confounding	Follow-up	Results					Practice implication
							Complete	Accurate	Believable	Applicable	Fit	
Bardes et al,^16^ 2017	Low	Low	Low	Low	Some concerns	Some concerns	Low	Some concerns	Some concerns	Low	Low	Some concerns
Boutonnet et al,^17^ 2018	Low	Low	High	Low	Low	Low	Low	Low	Low	Low	Low	Low
Chan et al,^18^ 2019	Low	Low	Low	Low	Low	Low	Low	Low	Low	Low	Low	Low
Cole et al,^19^ 2015	Low	Low	Low	Low	Low	Some	Low	Some concerns	Some concerns	Low	Low	Some concerns
Dixon et al,^20^ 2019	Low	Low	Low	Low	Some concerns	Some concerns	Low	Some concerns	Some concerns	Low	Low	Low
El-Menyar et al,^21^ 2020	Low	Low	Low	Low	Low	Some concerns	Low	Some concerns	Low	Low	Low	Low
Glover et al,^22^ 2019	High	Low	High	Low	High	Low	Low	Some concerns	Some concerns	Low	Low	Low
Harvin et al,^23^ 2015	Low	Low	High	Low	High	Low	Low	Low	Some concerns	Low	Low	Low
Howard et al,^24^ 2017	Low	Low	High	Low	Low	Low	Low	Some concerns	Some concerns	High	Low	Some concerns
Johnston et al,^25^ 2018	Low	Low	High	Low	High	Low	Low	Low	Some concerns	High	High	Low
Khan et al,^26^ 2018	Low	Low	High	Low	Low	Low	Low	Some concerns	Low	Low	Low	Low
Lewis et al,^27^ 2016	Low	Low	High	Low	High	Some	Low	Some concerns	High	High	Low	Some
Luehr et al,^28^ 2017	Low	Low	High	Some	High	Low	Low	Some concerns	Low	Low	High	Low
Moore et al,^29^ 2017	Low	Low	High	Low	High	Low	Low	Low	High	Low	Low	High
Morrison et al,^30^ 2012	Low	Low	Low	Low	High	Low	Low	Low	Some concerns	Low	Low	Low
Morrison et al,^31^ 2013	Low	Low	Low	Low	Low	Low	Low	Low	Low	High	Low	Low
Morte et al,^32^ 2019	Low	Low	Low	Low	Low	Low	Low	Low	Low	High	Low	Low
Myers et al,^33^ 2019	Low	Low	High	Low	Low	Some concerns	Low	Low	Some concerns	Low	High	Some concerns
Neeki et al,^34^ 2018	Low	Low	Low	Low	Low	Low	Low	Low	Low	Some concerns	Low	Some concerns
Neeki et al,^35^ 2020	Low	Low	Low	Low	Low	Low	Low	Low	Low	Some concerns	Low	Some concerns
Shiraishi et al,^36^ 2017	Low	Low	High	Low	Low	Low	Low	Low	Low	Some concerns	Low	Low
Swendsen et al,^37^ 2013	Low	Low	Some	Low	High	Low	Low	Low	Some concerns	Low	Low	Some concerns
Valle et al,^38^ 2014	Low	Low	Low	High	Low	Some concerns	Low	Low	Some concerns	Some concerns	High	Some concerns
Wafaisade et al,^39^ 2016	Low	Low	High	Low	Low	Low	Low	Low	Low	Low	Low	Low
Walker et al,^40^ 2020	Low	High	High	Low	High	Low	Low	Some concerns	Some concerns	High	High	Some concerns

^a^ Assessed using the Critical Appraisal Skills Programme.^7^ Each included study was scored on 12 aspects (from left to right): (1) whether the study addressed a clearly focused issue; (2) whether the cohort was chosen in an acceptable way; (3) whether the exposure was precisely measured to reduce bias; (4) whether the outcome was precisely measured to reduce bias; (5) whether the authors identified all significant confounding factors and whether they considered confounding factors in the design or analysis; (6) whether the follow-up of participants was complete and long enough; (7) whether the results of this study were complete; (8) whether the results were accurate; (9) whether the results were believable; (10) whether the results could be applied to local population; (11) whether the results fit with other available evidence; and (12) whether this study provided implications for practice.

### Association Between Tranexamic Acid Administration and Mortality in Patients With Traumatic Injury

#### Meta-analysis of 24-Hour Mortality Data

Data for the meta-analysis of 24-hour mortality was not pooled owing to significant heterogeneity (*I*^2^ = 85%; *P* < .001). All included studies except 1 reported mortality rates. Twenty-one studies^2,3,4,13,15,16,20,21,22,24,25,26,28,30,31,32,34,35,36,37,39^ had reported lower mortality and 9 studies^14,17,19,23,27,29,33,38,40^ had reported higher mortality for tranexamic acid treatment. A common quantitative measure would be misleading because the risk ratios (RRs) varied from 0.11 (95% CI, 0.01-0.80) to 3.38 (95% CI, 2.41-4.74). A sensitivity analysis of observational studies excluding studies with a high risk of bias due to confounding was performed, but tranexamic acid treatment with low heterogeneity was not associated with a survival advantage (RR, 0.67 [95% CI, 0.45-1.01]; *I*^2^ = 0%; 95% PI, 0.42-1.07). A sensitivity analysis of RCTs and subgroup analyses would not have been useful because each would have had included fewer than 5 studies.

#### Meta-analysis of 1-Month Mortality Data

Data for the meta-analysis of 1-month mortality was associated with a 17% decrease in mortality for tranexamic acid, with a pooled RR estimate of 0.83 (95% CI, 0.71-0.97) and a moderate heterogeneity (*I*^2^ = 35%; 95% PI, 0.55-1.27) (Figure 2). This was confirmed by a sensitivity analysis of RCTs with no heterogeneity (RR, 0.92 [95% CI, 0.87-0.97]; *I*^2^ = 0%; 95% PI, 0.87-0.97). The sensitivity analysis of observational studies excluding studies at high risk for confounding bias was not associated with greater survival benefits compared with the RCT analysis (RR, 0.73 [95% CI, 0.53-1.00]), and heterogeneity was moderate (*I*^2^ = 49%; 95% PI, 0.34-1.55).

#### Meta-analysis of Overall Mortality Data

Owing to significant heterogeneity (*P* < .001), data for overall mortality were not pooled. The RR estimates varied from 0.09 (95% CI, 0.01-1.60) to 2.94 (95% CI, 1.81-4.80). A sensitivity analysis for overall mortality rates among RCTs showed a decrease in mortality for tranexamic acid treatment with low heterogeneity (RR, 0.92 [95% CI, 0.86-0.98]; *I*^2^ = 0%; 95% PI, 0.86-0.99). The sensitivity analysis of observational studies was not performed owing to large heterogeneity (*I*^2^ = 78%; *P* < .001).

### Association Between Tranexamic Acid and Thromboembolic Events in Patients With Traumatic Injury

Data for thromboembolic events could not be pooled owing to significant heterogeneity (*I*^2^ = 73%; *P* < .001). Estimates of RRs varied from 0.14 (95% CI, 0.01-2.69) to 24.12 (95% CI, 1.42-408.88) (Figure 2). With the exception of 7 observational studies,^16,17,27,29,31,38,40^ all studies had reported thromboembolic events. Twelve studies^3,14,19,21,22,23,24,25,30,32,33,37^ revealed higher and 8 studies^2,4,13,18,20,28,36,39^ revealed lower incidences of thromboembolic events for tranexamic acid treatment compared with the control cohort. Three studies^26,34,35^ could not find any association (RR, 1.00) between tranexamic acid administration and thromboembolic events. It was not possible to resolve heterogeneity through the analysis of RCTs only (*I*^2^ = 59%; *P* = .03). In addition, a sensitivity analysis of observational studies excluding studies at high risk for confounding bias found a numerical increase of 31% in thromboembolic events compared with the control cohort, but imprecision was high (RR, 1.31 [95% CI, 0.91-1.88]; *I*^2^ = 7%; 95% PI, 0.81-2.12).

### Subgroups

Subgroup analyses did not alter the results significantly. Therefore, this information is presented in the eResults in the Supplement.

### Reporting Bias

The funnel plots were slightly asymmetric, suggesting a potential risk of publication bias. Small or medium studies appeared to be missing. The 1-month mortality funnel plot indicates lack of studies, with large RRs in favor of a higher mortality risk with the administration of tranexamic acid (eFigure 28 in the Supplement). The funnel plot for thromboembolic events indicates the opposite—that is, a lack of studies with lower RRs in favor of a lower risk for thromboembolic events with tranexamic acid administration (eFigure 29 in the Supplement).

## Discussion

This report details a systematic review and meta-analysis on the use of tranexamic acid in patients with traumatic injury. The main strength of this review is its size, including 31 studies that summarize, to our knowledge, all the clinical evidence published thus far. However, given the large diversity of the included trials, the results must be analyzed critically.

Overall, tranexamic acid administration was associated with a 17% decrease in 1-month mortality compared with the control group. A subgroup analysis revealed that patients with multiple traumatic injuries may benefit more from tranexamic acid administration than patients with TBI, in particular when the leading pathological finding was hemorrhage along with clinical signs of shock. Although the results for patients with TBI were mainly dominated by a single study (ie, the Clinical Randomisation of an Antifibrinolytic in Significant Head Injury [CRASH-3] study^13^), the comparison between the 2 entities may be viewed with caution. However, the present results are corroborated by the most recent RCT, in which results of comparing patients with nonsevere and severe TBI were largely similar.^3^

Although all meta-analyses for 1-month mortality were sufficiently homogenous, data pooling for 24-hour mortality, overall mortality, and thromboembolic events was not possible. Different approaches may explain major heterogeneity among studies. Methodological heterogeneity was addressed through sensitivity analyses for RCTs and observational studies that excluded studies with high risk of bias for confounders. After subsequent data pooling, both observational studies and RCTs showed decreased 24-hour mortality and overall mortality, respectively. The results of the sensitivity analysis of observational studies suggest that tranexamic acid administration may be associated with a 31% increase in thromboembolic events.

Different sources for clinical heterogeneity when analyzing potential reasons for large differences in study outcomes have been suggested.^41^ First, clinical heterogeneity may be based on patient characteristics.^41^ It is important to consider injury severity, which is directly related to higher odds of death.^42^ Eleven observational studies^16,19,21,22,23,25,28,29,30,37,40^ did not match intervention and control groups regarding patient baseline characteristics, which often led to significant differences in the ISS. In 2 studies,^23,29^ the ISS between intervention and control groups differed dramatically, because patients who had received tranexamic acid had more severe injury. The present results also confirm that in studies in which the intervention group was more severely injured, mortality along with tranexamic acid administration was higher. However, the meaningfulness of these possibly distorted results is questionable, and their impact on heterogeneity when pooling data for overall mortality needs to be considered. Information on age, sex, or ISS was missing in several RCTs^2,13,14^ and observational studies^16,17,18,35,40^; their impact on heterogeneity is thus unknown. Moreover, information on potential comorbidities was lacking. These may also correlate with both mortality and thromboembolic events; hence, the analysis of heterogeneity would have benefited from this information.^43,44^

Second, clinical heterogeneity may result from differences in the intervention itself.^41^ It was found that reporting of tranexamic acid dosages and timing was inconsistent. We aimed to address intervention differences in a post hoc subgroup analysis for each outcome. According to the CRASH-2 and CRASH-3 protocols,^7,13^ a bolus of 1 g of tranexamic acid followed by an infusion of 1 g of tranexamic acid was defined as standard. This regimen has been widely adopted clinically and corresponds to the recommendation given by the European Task Force for Advanced Bleeding Care in Trauma guideline.^45^ A distinction between standard and nonstandard tranexamic acid administration did not result in homogeneity in any of the heterogeneous outcomes. The survival benefit regarding 1-month mortality did not differ between standard and nonstandard administration. It may be concluded that survival in patients with bleeding traumatic injury depends less on how tranexamic acid is dosed and more on whether it is administered at all.

Third, outcome differences may contribute to clinical heterogeneity. Eight studies^13,14,19,20,22,23,25,37,46^ reported their approach while adhering to the guidelines for diagnosis and management of venous thromboembolism. The remaining studies did not report methods to assess thromboembolic events. Because the detection of thromboembolic events strongly depends on the sensitivity of the diagnostic tests performed, missing information may be considered as a factor contributing to heterogeneity of pooled data. Moreover, heterogeneity may be based on differences regarding the length of follow-up. The present study included follow-up from 72 hours^22^ to 6 months^4^ for the analysis of overall mortality. There was no association between length of follow-up and overall mortality, and this may be considered an irrelevant bias not contributing to heterogeneity. Follow-up times for thromboembolic events did not differ between studies.

Other variables that may influence clinical heterogeneity are differences in research settings, overall clinical management, including timely access to standard critical care, and geographical issues. Twenty-nine studies from 10 different countries as well as 2 multinational studies led to large differences in clinical conditions and supposedly management of traumatic injury. Because tranexamic acid may limit acute traumatic bleeding, it may be more valuable in environments where patients are treated without rapid access to advanced care, including blood products and other hemostatic agents. For example, in the CRASH-2 study,^2^ fewer than 2% of patients had been treated in countries that routinely provide rapid access to blood products, damage-control surgery, and advanced critical care.^47^ The Prehospital Antifibrinolytics for Traumatic Coagulopathy and Haemorrhage trauma trial^48^ is currently assessing the CRASH-2 protocol in advanced trauma care systems in Australia, New Zealand, and Germany, and results are expected shortly.

Critical reporting and sufficient analyses are crucial when it comes to investigating heterogeneity of meta-analyses in systematic reviews, and corresponding guidelines such as the *Cochrane Handbook for Systematic Reviews of Interventions* should be respected.^5,8^ Failure to fully reflect heterogeneity of results may lead to misinterpretations, incorrect assumptions, and incorrect and potentially risky clinical recommendations. Methodologically high-quality reviews are rarely found in the areas of trauma care and emergency surgery, and reported results should be interpreted with caution. Although previous systematic reviews^49,50,51,52,53,54^ have consistently reported mortality risk reduction with tranexamic acid after trauma, there remain discrepancies regarding thromboembolic events. Significant heterogeneity in the report of thromboembolic events between the studies was observed in the present study that led to large PIs or to results that precluded data pooling. Only sensitivity analysis of observational studies after exclusion of studies with high risk of bias from confounders revealed that tranexamic acid is associated with an increased risk for thromboembolic events. These findings are in contrast to other systematic reviews^51,52,54^ that have reported decreased risk of thromboembolic events. One recent review^51^ did not consider χ^2^ tests indicating significant heterogeneity in their meta-analysis, which should have led to the assumption that data cannot be pooled. Another review^52^ did not report any tests for heterogeneity in respect to their included population with traumatic injury, rendering the results uninterpretable. A popular way of expressing heterogeneity in a meta-analysis is the use of PIs.^55^ A 95% PI estimates where the true effects can be expected for 95% of similar studies that might be conducted in the future and is often more informative than the *I*^2^ value.^56^ The Cochrane guidelines recommend the use of PIs.^8^ Several reviewers^8,49,50,51,52,53,54,57^ who reported no differences in thromboembolic events between intervention and control cohorts have implemented heterogeneity assessments only in part, rendering their results questionable.

### Limitations

This systematic review has some limitations. The search strategy only included literature published in English and German without a search for gray literature. Asymmetrical funnel plots indicate publication bias. Observational studies usually have a low internal validity owing to their risk of bias and therefore may distort results. It was aimed to resolve these methodological differences through sensitivity analyses. Moreover, several studies did not report information on baseline characteristics, comorbidities, tranexamic acid dosage regimen, and follow-up times. Despite being fully analyzed in the discussion, their impact on the results remains unclear.

## Conclusions

This systematic review and meta-analysis found that tranexamic acid was associated with a 17% decrease in 1-month mortality in patients with traumatic injury compared with a control cohort. Reasonable concerns about potential thromboembolic events with tranexamic acid remain, but a definitive conclusion cannot be reached owing to a lack of homogenous data. Therefore, the use of tranexamic acid, as with any other pharmacological therapy, needs to be balanced against its potential risks. Systematic reviews constitute a complex research tool that can have a powerful impact on future decision making; however, it is essential to fully implement guidelines for systematic reviews to ensure that results are well generated and analyzed. When heterogeneity occurs, it needs to be reported sufficiently while evaluating population characteristics, study interventions, and outcome assessments.

## Notes

Supplement. **eMethods.** Search Strategy, Selection Process, and Data Items Extracted **eResults.** Study Selection, Study Characteristics, Outcomes, and Results of Subgroup Analyses **eFigure 1.** PRISMA Flow Diagram **eTable 1.** List of Excluded Studies With Reasons **eTable 2.** Data Extraction Sheet **eTable 3.** Extracted Data of Blood Transfusions **eFigure 2.** 24-Hour Mortality **eFigure 3.** 24-Hour Mortality in Observational Studies Excluding Studies at High Risk for Confounding Bias **eFigure 4.** 1-Month Mortality in RCTs **eFigure 5.** 1-Month Mortality in Observational Studies Excluding Studies at High Risk for Confounding Bias **eFigure 6.** 1-Month Mortality in Multiple Trauma **eFigure 7.** 1-Month Mortality in TBI **eFigure 8.** 1-Month Mortality in Standard Protocol Tranexamic Acid Administration **eFigure 9.** 1-Month Mortality in Nonstandard Protocol Tranexamic Acid Administration **eFigure 10.** Overall Mortality **eFigure 11.** Overall Mortality in RCTs **eFigure 12.** Overall Mortality in Observational Studies Excluding Studies at High Risk for Confounding Bias **eFigure 13.** Overall Mortality in Multiple Trauma **eFigure 14.** Overall Mortality in TBI **eFigure 15.** Overall Mortality in In-hospital Tranexamic Acid Administration **eFigure 16.** Overall Mortality in Prehospital Tranexamic Acid Administration **eFigure 17.** Overall Mortality in Standard Protocol Tranexamic Acid Administration **eFigure 18.** Overall Mortality in Nonstandard Tranexamic Acid Administration **eFigure 19.** Thromboembolic Events **eFigure 20.** Thromboembolic Events in RCTs **eFigure 21.** Thromboembolic Events in Observational Studies Excluding Studies at High Risk for Confounding Bias **eFigure 22.** Thromboembolic Events in Multiple Trauma **eFigure 23.** Thromboembolic Events in TBI **eFigure 24.** Thromboembolic Events in In-hospital Tranexamic Acid Administration **eFigure 25.** Thromboembolic Events in Prehospital Tranexamic Acid Administration **eFigure 26.** Thromboembolic Events in Standard Protocol Tranexamic Acid Administration **eFigure 27.** Thromboembolic Events in Nonstandard Protocol Tranexamic Acid Administration **eFigure 28.** Funnel Plot for 1-Month Mortality **eFigure 29.** Funnel Plot for Thromboembolic Events
eReferences
