# Supplementary material for: Association of Tranexamic Acid Administration With Mortality and Thromboembolic Events in Patients With Traumatic Injury: A Systematic Review and Meta-analysis
Source: JAMA Netw Open. 2022 Mar 1;5(3):e220625. doi: 10.1001/jamanetworkopen.2022.0625 (PMC8889461; doi:10.1001/jamanetworkopen.2022.0625)
Supplement: Supplement. — eMethods. Search Strategy, Selection Process, and Data Items Extracted eResults. Study Selection, Study Characteristics, Outcomes, and Results of Subgroup Analyses eFigure 1. PRISMA Flow Diagram eTable 1. List of Excluded Studies With Reasons eTable 2. Data Extraction Sheet eTable 3. Extracted Data of Blood Transfusions eFigure 2. 24-Hour Mortality eFigure 3. 24-Hour Mortality in Observational Studies Excluding Studies at High Risk for Confounding Bias eFigure 4. 1-Month Mortality in RCTs eFigure 5. 1-Month Mortality in Observational Studies Excluding Studies at High Risk for Confounding Bias eFigure 6. 1-Month Mortality in Multiple Trauma eFigure 7. 1-Month Mortality in TBI eFigure 8. 1-Month Mortality in Standard Protocol Tranexamic Acid Administration eFigure 9. 1-Month Mortality in Nonstandard Protocol Tranexamic Acid Administration eFigure 10. Overall Mortality eFigure 11. Overall Mortality in RCTs eFigure 12. Overall Mortality in Observational Studies Excluding Studies at High Risk for Confounding Bias eFigure 13. Overall Mortality in Multiple Trauma eFigure 14. Overall Mortality in TBI eFigure 15. Overall Mortality in In-hospital Tranexamic Acid Administration eFigure 16. Overall Mortality in Prehospital Tranexamic Acid Administration eFigure 17. Overall Mortality in Standard Protocol Tranexamic Acid Administration eFigure 18. Overall Mortality in Nonstandard Tranexamic Acid Administration eFigure 19. Thromboembolic Events eFigure 20. Thromboembolic Events in RCTs eFigure 21. Thromboembolic Events in Observational Studies Excluding Studies at High Risk for Confounding Bias eFigure 22. Thromboembolic Events in Multiple Trauma eFigure 23. Thromboembolic Events in TBI eFigure 24. Thromboembolic Events in In-hospital Tranexamic Acid Administration eFigure 25. Thromboembolic Events in Prehospital Tranexamic Acid Administration eFigure 26. Thromboembolic Events in Standard Protocol Tranexamic Acid Administration eFigure 27. Thromboembolic Events in Nonstandard Prot [file jamanetwopen-e220625-s001.pdf]

Supplementary Online Content

Karl V, Thorn S, Mathes T, Hess S, Maegele M. Association of tranexamic acid administration with mortality and thromboembolic events in patients with traumatic injury: a systematic review and meta-analysis. *JAMA Netw Open*. 2022;5(3):e220625. doi:10.1001/jamanetworkopen.2022.0625

eMethods. Search Strategy, Selection Process and Data Items Extracted

eResults. Study Selection, Study Characteristics, Outcomes, and Results of Subgroup Analyses

eFigure 1. PRISMA Flow Diagram

eTable 1. List of Excluded Studies With Reasons

eTable 2. Data Extraction Sheet

eTable 3. Extracted Data of Blood Transfusions

eFigure 2. 24-Hour Mortality

eFigure 3. 24-Hour Mortality in Observational Studies Excluding Studies at High Risk for Confounding Bias

eFigure 4. 1-Month Mortality in RCTs

eFigure 5. 1-Month Mortality in Observational Studies Excluding Studies at High Risk for Confounding Bias

eFigure 6. 1-Month Mortality in Multiple Trauma

eFigure 7. 1-Month Mortality in TBI

eFigure 8. 1-Month Mortality in Standard Protocol Tranexamic Acid Administration

eFigure 9. 1-Month Mortality in Nonstandard Protocol Tranexamic Acid Administration

eFigure 10. Overall Mortality

eFigure 11. Overall Mortality in RCTs

eFigure 12. Overall Mortality in Observational Studies Excluding Studies at High Risk for Confounding Bias

eFigure 13. Overall Mortality in Multiple Trauma

eFigure 14. Overall Mortality in TBI

eFigure 15. Overall Mortality in In-hospital Tranexamic Acid Administration

eFigure 16. Overall Mortality in Prehospital Tranexamic Acid Administration

eFigure 17. Overall Mortality in Standard Protocol Tranexamic Acid Administration

eFigure 18. Overall Mortality in Nonstandard Tranexamic Acid Administration

eFigure 19. Thromboembolic Events

eFigure 20. Thromboembolic Events in RCTs

eFigure 21. Thromboembolic Events in Observational Studies Excluding Studies at High Risk for Confounding Bias

eFigure 22. Thromboembolic Events in Multiple Trauma

eFigure 23. Thromboembolic Events in TBI

eFigure 24. Thromboembolic Events in In-hospital Tranexamic Acid Administration

eFigure 25. Thromboembolic Events in Prehospital Tranexamic Acid Administration

© 2022 Karl V et al. *JAMA Network Open*.

eFigure 26. Thromboembolic Events in Standard Protocol Tranexamic Acid Administration

eFigure 27. Thromboembolic Events in Nonstandard Protocol Tranexamic Acid Administration

eFigure 28. Funnel Plot for 1-Month Mortality

eFigure 29. Funnel Plot for Thromboembolic Events

eReferences

This supplementary material has been provided by the authors to give readers additional information about their work.

© 2022 Karl V et al. *JAMA Network Open*.

eMethods. Search Strategy, Selection Process and Data Items Extracted

Search strategy

The search strategy was prepared by an experienced information specialist in collaboration with clinical experts. The search was limited to English and German language. We searched for studies published from 1986 (when TXA was approved for medical use) onwards. We excluded in vitro studies and animal experiments. The search strategy was based on the Peer Review of Electronic Search Strategies checklist (PRESS) and validated by checking if pre-defined studies that are clearly eligible would have been identified [1]. The detailed search strategy is shown below.

Medline via Ovid (Ovid MEDLINE(R) and Epub Ahead of Print, In-Process & Other Non-Indexed Citations, Daily And Version(R))

1 *Craniocerebral Trauma/ or *brain injuries/ or exp brain injuries, traumatic/ or exp head injuries, closed/ or exp head injuries, penetrating/ or exp skull fractures/ 94218

2 (craniocerebral trauma* or skull fracture* or traumatic brain injur* or tbi or ((head or brain) adj (injur* or trauma*))).ti,ab,kf. 105128

3 exp *"Wounds and Injuries"/ 744665

4 (injur* or trauma*).ti,ab,kf 1081710

5 1 or 2 or 3 or 4 1486642

6 exp Tranexamic Acid/ 3675

7 (tranexamic acid or txa).ti,ab,kf. 5579

8 6 or 7 6351

9 5 and 8 1073

10 (english or german).lg. 27969887

11 9 and 10 1018

12 exp animals/ not humans.sh. 4760027

13 11 not 12 885

14 (comment or editorial or letter).pt. 1905379

15 13 not 14 810

16 limit 15 to yr=1986-2021 791

Embase via Embase

#1 'head injury'/exp OR 'brain injury'/mj OR 'traumatic brain injury'/mj OR 'skull fracture'/exp 319,284

#2 ("craniocerebral trauma*" OR "skull fracture*" OR "traumatic brain injur*" OR tbi OR ((head OR brain) NEAR/1 (injur* OR trauma*))):ti,ab,kw 151,460

#3 'injury'/exp 2,401,780

#4 (injur* OR trauma*):ti,ab,kw 1,406,552

#5 tranexamic acid'/exp OR ("tranexamic acid" OR txa):ti,ab,kw 18,874

#6 (english OR german):la 33,439,122

#7 ('animals'/exp NOT 'humans'/de) 5,680,425

#8 (comment OR editorial OR letter):it 1,800,517

© 2022 Karl V et al. *JAMA Network Open*.

#9 [embase]/lim 26,606,377

#10 embase NOT (embase AND medline) 8,264,585

#11 [1986-2021]/py 30,346,918

#12 #1 OR #2 OR #3 OR #4 2,910,88

#13 #5 AND #12 4,060

#14 #6 AND #13 3,886

#15 #14 NOT #7 3,367

#16 #15 NOT #8 3,141

#17 #9 AND #16 2,963

#18 #10 AND #17 853

#19 #11 AND #18 844

#20 #19 AND ('article'/it OR 'article in press'/it OR 'erratum'/it OR 'review'/it) 480

Cochrane Libraray via Cochrane

#1 MeSH descriptor: [Craniocerebral Trauma] this term only 335

#2 MeSH descriptor: [Brain Injuries] this term only 1857

#3 MeSH descriptor: [Brain Injuries, Traumatic] explode all trees 780

#4 MeSH descriptor: [Head Injuries, Closed] explode all trees 422

#5 MeSH descriptor: [Head Injuries, Penetrating] explode all trees 2

#6 MeSH descriptor: [Skull Fractures] explode all trees 267

#7 (craniocerebral trauma* or skull fracture* or traumatic brain injur* or tbi or ((head or brain) AND (injur* or trauma*))):ti,ab,kw 12477

#8 MeSH descriptor: [Wounds and Injuries] explode all trees 25464

#9 (injur* or trauma*):ti,ab,kw 73143

#10 #1 OR #2 OR #3 OR #4 OR #5 OR #6 OR #7 OR #8 OR #9 82828

#11 MeSH descriptor: [Tranexamic Acid] explode all trees 1041

#12 (tranexamic acid or txa):ti,ab,kw 2844

#13 #11 OR #12 2844

#14 #10 AND #13 with Cochrane Library publication date Between Jan 1986 and Mar 2021 369

#15 (clinicaltrials.gov OR ICTRP OR Cinahl):so 201022

#16 #14 NOT #15 275

Selection process

Titles and abstracts of records identified by the electronic literature search were screened first. Next, full text articles were retrieved and screened for eligibility criteria. Disagreements were solved through discussion between the two reviewers or after consultation with a third or fourth reviewer until consensus. Multiple reports of the same study were merged so that each study is the unit of analyses.

Data items extracted

© 2022 Karl V et al. *JAMA Network Open*.

We extracted data of the latest available follow-up time for the outcomes overall mortality and thromboembolic events. As there are some studies which conducted a subgroup analysis within their trials, there are cases in which we extracted different data from the same study according to the subgroup. Missing data were marked as such and evaluated in the bias assessment and discussion, respectively. Extracted data items were extracted as follows.

1. bibliometric information:

1.1. name of all author

1.2 name of the Journal

1.2. year of publication

2. trial-specific information:

2.1. number and geographic regions of recruiting centers

2.2. NCT number, i.e., unique identification code given to each clinical study record registered on ClinicalTrials.gov

2.3. enrollment start and end dates

2.4. length of participant follow-up

3. participant-specific information:

3.1. number of randomized participants

3.2. detailed inclusion and exclusion criteria

3.3. baseline characteristics of participants such as age and gender

3.4. relevant clinical characteristics such as vital signs and ISS

3.5. Patient ratios of blunt vs. penetrating and TBI vs. multiple trauma

4. intervention(s) and comparison intervention(s)

4.1. medication

4.2. doses

4.3. frequency

5. outcomes:

5.1. mortality at 24 hours, 28 and 30 days, respectively as well as overall mortality

5.2. thromboembolic events

amount of blood transfusion given

5.3. length of follow-up (if necessary for each outcome separately)

6. subgroup allocation

6.1. multiple trauma including TBI vs predominantly TBI

6.2. severely vs. non-severely injured

6.3. blunt vs. penetrating trauma

6.4. inhospital vs. prehospital TXA administration

6.5. administration of TXA within 3 hours of onjury vs. beyond 3 hours of injury

© 2022 Karl V et al. *JAMA Network Open*.

eResults. Study Selection, Study Characteristics, Outcomes, and Results of Subgroup Analyses

Study selection

Our systematic literature search identified 1546 records. Two additional studies were detected through manual reference screening of related articles. After removal of duplicates, 1317 abstracts were screened for inclusion and exclusion criteria. 71 publications were included for full text screening, at which stage 40 studies were excluded (eTable 1). We included 31 studies in the systematic review.

Study Characteristics

Seven studies included trauma patients presenting predominantly with traumatic brain injury[7-13] while 24 studies included multiple trauma patients[14-37]. Five studies included trauma patients receiving prehospital TXA[9,15,20,32,37] and 6 studies described that TXA was first administered in-hospital[7,8,10,14,33]. The studies were conducted in the United States[9,12,13,15,16,19,22-28,31-33,35,36], United Kingdom[18,21,29,30], Canada[9], Germany[37], France[17], China[11], Japan[34], Tunisia[7], Thailand[10], Qatar[20] and worldwide[8,14].

Outcomes

During study selection it became apparent that some studies did not report the time at which mortality was measured (follow-up time), whereas others reported follow-up times that differed from the above. In order to avoid excluding many studies due to incorrect follow-up times, we suggested overall mortality in addition. This outcome encompassed mortality of all aforementioned time points plus mortaltiy for which no time frame was given.

It was not possible to conduct a meta-analysis for the amount of blood products given. A lot of data on blood product transfusion were reported as median instead of mean. Moreover, due to major clinical differences between the studies regarding trauma severity, data varied largely and led to significant heterogeneity. Thus, we had to discard the outcome amount of blood products given. Detailed data of blood products given can be seen in the Supplement XIII.

Results of subgroup analyses

After extracting data it was impossible to conduct meta analyses of some subgroups. First, most of the studies included patients that were severely injured, therefore we did not have sufficient data for the non-severely injured cohort to compare. Second, we are missing studies that compare blunt vs. penetrating trauma and TXA administration within 3 hours vs. beyond 3 hours, respectively. Finally, we conducted meta-analyses of the subgroups multiple trauma vs. TBI and in-hospital vs. prehospital TXA administration. For the latter, data availability limited our analysis to the outcomes overall mortality and thromboembolic events.

After recognising that most of our results were statistically heterogenous, we hypothesized that different TXA dosage regimens between trials would be a reason. Some included studies did not define a dosage regimen prior to study initiation while others applied a dosage regimen that differs from the The European guideline on management of major bleeding and coagulopathy following trauma: 1g IV bolus, followed by 1g IV maintenance dose over 8 hours [38]. Therefore we conducted post-hoc meta-analyses comparing standard TXA administration and non-standard administration.

Meta-analysis of one-month mortality

A subgroup analysis of the different types of trauma suggested a greater effect in the TXA groups of multiple trauma patients (RR = 0.77, 95% CI = 0.63-0.94, I^2 = 43%, 95% PI = 0.48 – 1.25) than of TBI patients (RR = 0.94, 95% CI = 0.85-1.03, I^2 = 0%, 95% PI = 0.83 – 1.06). Notably, almost 90% of the results of the TBI subgroup analysis were determined by the CRASH-3 study. The post-hoc subgroup analysis regarding TXA standard administration, defined as 1g bolus plus 1g maintenance dose, and non-standard administration resulted in a similar RR of 0.82 while the standard administration was accompanied by larger heterogeneity (95% CI = 0.58 – 1.16, I^2 = 50%, 95% PI = 0.33 – 2.03) than the non-standard administration (95% CI = 0.69 – 0.97, I^2 = 0%, 95% PI = 0.69 – 0.98).

© 2022 Karl V et al. *JAMA Network Open*.

Meta-analysis of overall mortality

To explore heterogeneity we performed a subgroup analysis. For multiple trauma patients we could not resolve heterogeneity ($p < 0.01$). RR estimates varied between 0.21 and 2.94. The analysis of TBI patients showed that TXA administration was associated with reduction in overall mortality (RR = 0.94, 95% CI = 0.86-1.03, I^2 = 0%, 95% PI = 0.86 – 1.03). Prehospital and in-hospital administration of TXA both reduced overall mortality rates with the same estimated RR of 0.82. Thereby, the prehospital subgroup showed a smaller confidence interval and lower heterogeneity (95% CI = 0.61-1.10, I^2 = 15%, 95 % PI = 0.49 – 1.36) compared to the in-hospital subgroup (95% CI = 0.44-1.54, I^2 = 48%, 95% PI = 0.17 – 3.89). Data of the post-hoc subgroup analysis of TXA standard administration and non-standard administration were not pooled because heterogeneity was significant in both cases ($p = 0.04$ / $p < 0.01$).

Meta-analysis of thromboembolic events

A subgroup analysis of TBI patients showed that thromboembolic events decreased when TXA was administered. However, the prediction interval that effect estimates in future studies may vary widely (RR = 0.91, 95% CI = 0.43-1.91, I^2 = 44%, 95% PI= 0.19 – 4.31). Data of the multiple trauma subgroup was not pooled because of heterogeneity ($p < 0.01$). In-hospital TXA administration suggested that TXA may decrease incidence of thromboembolic events (RR = 0.93, 95% CI = 0.51-1.72, I^2 = 35%, 95% PI = 0.27 – 3.27), while prehospital TXA administration increase thromboembolic events negligibly (RR = 1.01, 95% CI = 0.53-1.94, $p = 0.14$, I^2 = 43%, 95% PI = 0.32 – 3.18). Statitistical heterogenity was high for both comparisons. A post-hoc subgroup analysis regarding standard administration and non-standard administration did not resolve this heterogeneity ($p < 0.01$ / $p < 0.01$).

© 2022 Karl V et al. *JAMA Network Open*.

eFigure 1. **PRISMA flow diagram**

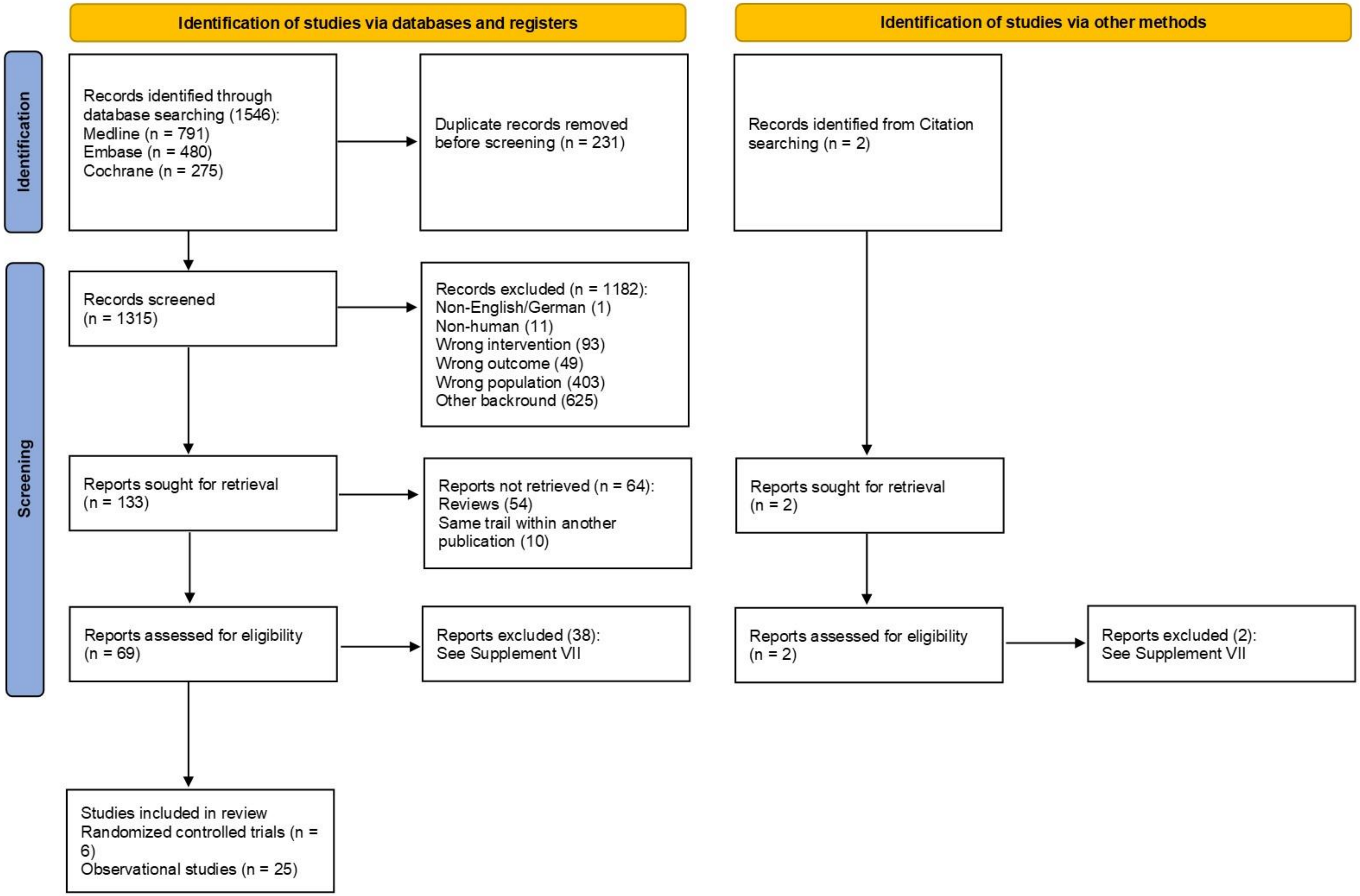

© 2022 Karl V et al. *JAMA Network Open*.

eTable 1. **List of Excluded Studies With Reasons**

Dixon AL, McCully BH, Rick EA, Dewey E, Farrell DH, Morrison LJ, et al. Tranexamic acid administration in the field does not affect admission thromboelastography after traumatic brain injury. The Journal of Trauma and Acute Care Surgery. 2020;89(5):900-7.	O
Ebrahimi P, Mozafari J, Ilkhchi RB, Hanafi MG, Mousavinejad M. Intravenous Tranexamic Acid for Subdural and Epidural Intracranial Hemorrhage: Randomized, Double-Blind, Placebo-Controlled Trial. Reviews on Recent Clinical Trials. 2019;14(4):286-91.	O
Fakharian E, Abedzadeh-Kalahroudi M, Atoof F. Effect of Tranexamic Acid on Prevention of Hemorrhagic Mass Growth in Patients with Traumatic Brain Injury. World Neurosurgery. 2018;109:e748-e53.	P
May S, Prehospital Tranexamic Acid Use for Traumatic Brain Injury (TXA). 2019.	D
Meretoja A, Yassi N, Wu TY, Churilov L, Sibolt G, Jeng JS, et al. Tranexamic acid in patients with intracerebral haemorrhage (STOP-AUST): a multicentre, randomised, placebo-controlled, phase 2 trial. Lancet Neurology. 2020;19(12):980-7.	P
Monsef Kasmaei V, Javadi A, Naseri Alavi SA. Effects of tranexamic acid on reducing blood loss in pelvic trauma: A randomised double-blind placebo controlled study. Journal of Clinical Orthopaedics & Trauma. 2019;10(2):286-9.	P
Schreiber MA. Prehospital tranexamic acid improves survival after traumatic brain injury in patients with intracranial hemorrhage. Shock (Augusta, Ga). 2019;51(6):26.	D
Sperry J. Study of Tranexamic Acid During Air Medical Prehospital Transport Trial (STAAMP Trial) (STAAMP).	D
Acar N, Canakci ME, Bilge U. Early and Ultraearly Administration of Tranexamic Acid in Traumatic Brain Injury: Our 8-Year-Long Clinical Experience. Emergency Medicine International Print. 2020;2020:6593172.	C
Adair KE, Patrick JD, Kliber EJ, Peterson MN, Holland SR. TXA (Tranexamic Acid) Risk Evaluation in Combat Casualties (TRECC). Trauma Surgery & Acute Care Open. 2020;5(1):e000353.	U
Balvers K, van Dieren S, Baksaas-Aasen K, Gaarder C, Brohi K, Eaglestone S, et al. Combined effect of therapeutic strategies for bleeding injury on early survival, transfusion needs and correction of coagulopathy. British Journal of Surgery. 2017;104(3):222-9.	U
Blackwell JR, Leggett T, Kukadia J, Sahemey R, Lopes R, Nasir H, et al. Venous thrombo-embolism following tranexamic acid in the trauma patient. Trauma (united kingdom) Conference: 17th international trauma care conference United kingdom. 2016;18(4):318.	P

© 2022 Karl V et al. *JAMA Network Open*.

Boudreau RM, Deshpande KK, Day GM, Hinckley WR, Harger N, Pritts TA, et al. Prehospital Tranexamic Acid Administration During Aeromedical Transport After Injury. Journal of Surgical Research. 2019;233:132-8.	C
Chapman N. Use of tranexamic acid in trauma patients requiring massive transfusion protocol activation: an audit in a major trauma centre in New Zealand. New Zealand Medical Journal. 2018;131(1483):8-12.	O
Cornelius BG, McCarty K, Hylan K, Cornelius A, Carter K, Smith KWG, et al. Tranexamic Acid: Promise or Panacea: The Impact of Air Medical Administration of Tranexamic Acid on Morbidity, Mortality, and Length of Stay. Advanced Emergency Nursing Journal. 2018;40(1):27-35.	O
Cornelius B, Moody K, Hopper K, Kilgore P, Cvek U, Trutschl M, et al. A Retrospective Study of Transfusion Requirements in Trauma Patients Receiving Tranexamic Acid. Journal of Trauma Nursing. 2019;26(3):128-33.	O
David JS, Lambert A, Bouzat P, Incagnoli P, Geay-Baillat AM, Taverna XJ, et al. Fibrinolytic shutdown diagnosed with rotational thromboelastometry represents a moderate form of coagulopathy associated with transfusion requirement and mortality: A retrospective analysis. European Journal of Anaesthesiology. 2020;37(3):170-9.	O
DeHoff K, Milligan J, Zimmerman LH, Jurado L, Nakajima S, Acquista E. Impact of tranexamic acid on transfusions prevs post-protocol implementation in trauma patients. Critical care medicine Conference: 46th critical care congress of the society of critical care medicine, SCCM 2016 United states Conference start: 20170121 Conference end: 20170125. 2016;44(12 Supplement 1):461.	O
Douglas A, Faviere D, Gallo A, VanderSchaaf A, Wall W, Wilson K, et al. Impact of Early Tranexamic Acid Administration on Overall Blood Product Utilization at 24 Hours. Journal of Trauma Nursing. 2020;27(2):66-70.	C
Erramouspe PJ, Garcia-Pintos MF, Benipal S, Manoukian MAC, Santamarina JL, Shawagga HG, et al. Mortality and Complication Rates in Adult Trauma Patients Receiving Tranexamic Acid: A Single-center Experience in the Post-CRASH-2 Era. Academic Emergency Medicine. 2020;27(5):358-65.	C
Gall L, Davenport R, Brohi K. Effect of early tranexamic acid on the coagulation system in patients with suspected traumatic haemorrhage: a prospective cohort study. 2016.	F
Fernandez M, Morrison C, Pettineo G. Pilot study: the use of tranexamic acid in trauma patients at a level one teaching hospital. Crit Care Med. 2012.	C
Ghazi S, Pierson R, Torlinski T. Military application of tranexamic acid in trauma emergency resuscitation study 2C03, 3C00. Journal of the Intensive Care Society. 2013;14(1):86-8.	A
Guth C, Vassal O, Friggeri A, Wey PF, Inaba K, Decullier E, et al. Effects of modification of trauma bleeding management: A before and after study. Anaesthesia	C

Critical Care & Pain Medicine. 2019;38(5):469-76.	
Jachetti A, Massenat RB, Edema N, Woolley SC, Benedetti G, Van Den Bergh R, et al. Introduction of a standardised protocol, including systematic use of tranexamic acid, for management of severe adult trauma patients in a low-resource setting: the MSF experience from Port-au-Prince, Haiti. BMC Emergency Medicine. 2019;19(1):56.	I
Kheirbek T, Jikaria N, Murray B, Martin TJ, Lueckel SN, Stephen AH, et al. Unjustified Administration in Liberal Use of Tranexamic Acid in Trauma Resuscitation. Journal of Surgical Research. 2020;258:125-31.	C
Kunze-Szikszay N, Krack LA, Wildenauer P, Wand S, Heyne T, Walliser K, et al. The pre-hospital administration of tranexamic acid to patients with multiple injuries and its effects on rotational thrombelastometry: a prospective observational study in pre-hospital emergency medicine. Scandinavian Journal of Trauma, Resuscitation & Emergency Medicine. 2016;24(1):122.	C
Lawson K, Godfrey P. TXA for HEMS patients with suspected haemorrhage. 2017.	C
Lipsky AM, Abramovich A, Nadler R, Feinstein U, Shaked G, Kreiss Y, et al. Tranexamic acid in the prehospital setting: Israel Defense Forces' initial experience. Injury. 2014;45(1):66-70.	C
Marsden MER, Rossetto A, Duffield CAB, Woolley TGD, Buxton WP, Steynberg S, et al. Prehospital tranexamic acid shortens the interval to administration by half in Major Trauma Networks: a service evaluation. Emergency Medicine Journal. 2019;36(7):395-400.	P
Meizoso JP, Dudaryk R, Mulder MB, Ray JJ, Karcutskie CA, Eidelson SA, et al. Increased risk of fibrinolysis shutdown among severely injured trauma patients receiving tranexamic acid. The Journal of Trauma and Acute Care Surgery. 2018;84(3):426-32.	P
Milligan J, DeHoff K, Jurado L, Zimmerman LH, Nakajima S, Acquista E. Effects of early versus late tranexamic acid administration in critically ill trauma patients. 2016.	F
Morrison JJ, Ross JD, Dubose JJ, Jansen JO, Midwinter MJ, Rasmussen TE. Association of cryoprecipitate and tranexamic acid with improved survival following wartime injury: findings from the MATTERs II Study. JAMA Surgery. 2013;148(3):218-25.	I
Neeki MM, Dong F, Toy J, Vaezazizi R, Powell J, Jabourian N, et al. Efficacy and Safety of Tranexamic Acid in Prehospital Traumatic Hemorrhagic Shock: Outcomes of the Cal-PAT Study. The Western Journal of Emergency Medicine. 2017;18(4):673-83.	D
Nelson Yap KB, Albert Wong SH, Idris Z. Tranexamic acid in traumatic brain injury. Medical Journal of Malaysia. 2020;75(6):660-5.	P

© 2022 Karl V et al. *JAMA Network Open*.

Reed MJ, Cooke C, McMahon N, Hands K, Henderson S, Knight E, et al. Improvements in National Code Red transfusion practice in Scotland after adoption of recommendations from the Scottish National Code Red 2015 review. Injury. 2020;51(4):913-8.	I
Tencza E, Harrell AJ, Sarangarm P. Effect of tranexamic acid administration time on blood product use in urban trauma patients. American Journal of Health-System Pharmacy. 2020;77(Supplement_2):S46-S53.	C
Van Haren RM, Valle EJ, Thorson CM, Jouria JM, Busko AM, Guarch GA, et al. Hypercoagulability and other risk factors in trauma intensive care unit patients with venous thromboembolism. The Journal of Trauma and Acute Care Surgery. 2014;76(2):443-9.	P
Vu EN, Schlamp RS, Wand RT, Kleine-Deters GA, Vu MP, Tallon JM. Prehospital use of tranexamic acid for hemorrhagic shock in primary and secondary air medical evacuation. Air Medical Journal. 2013;32(5):289-92.	C
Walker PF, Schobel S, Caruso JD, Rodriguez CJ, Bradley MJ, Elster EA, et al. Trauma Embolic Scoring System in military trauma: a sensitive predictor of venous thromboembolism. Trauma Surgery & Acute Care Open. 2019;4(1):e000367.	O

P: wrong patients; I: wrong intervention; O: no relevant outcome; C: Missing control group; F: No full publication; A: Appraisal; U: data not usable, excluded during data extraction; D: Duplicate, Trial within another publication

© 2022 Karl V et al. *JAMA Network Open*.

eTable 2. **Data Extraction Sheet**

**Study/Reference**	**Region, setting, inclusion criteria, exclusion criteria and baseline characteristics (IG/CG) of study population**	**Intervention(s), control, patient flow (IG/CG) and length of follow-up period**
Chakroun-Walha O, Samet A, Jerbi M, Nasri A, Talbi A, Kanoun H, et al. Benefits of the tranexamic acid in head trauma with no extracranial bleeding: a prospective follow-up of 180 patients. European Journal of Trauma & Emergency Surgery. 2019;45(4):719-26.	**Region/Setting**<br>Tunisia<br>August 2016 – September 2017<br>**Inclusion criteria**<br>● Age ≥ 18 years<br>● Admitted to the ED for TBI<br>● Intracranial bleeding in the first or the second brain CT-scan, and with a delay of management in the study center under 24 h after trauma<br>**Exclusion criteria**<br>● Significant extra cranial bleeding (that is not in need of immediate blood transfusion)<br>● Patients with evidence that TXA improve outcome<br>● Known renal failure<br>● History or current evidence suggestive of venous or arterial thrombosis<br>**Patient characteristics IG/CG**<br><u>Age, years, mean(SD)</u><br>44 (20) / 39 (18)<br>**Gender**<br>M/F sex ratio 11/8.3<br><u>Vital signs</u><br>Shock defined as:<br>• Heart rate, bpm, mean(SD) 87 (16) / 86 (19)	**Intervention**<br>TXA bolus 1g, 10 min, maintenance 1g, 8h<br>**Control**<br>None<br>**Time of TXA administration after injury**<br>N/A<br>**Randomized patients**<br>96/84<br>**Follow-up**<br>28 days

© 2022 Karl V et al. *JAMA Network Open*.

	If TBI: GCS score, points, mean(SD) 9 (5) / 10 (5)<br><br><u>Injury Severity Score, points, mean(SD)</u><br><br>21.8 (23.2) / 23.5 (25.6)<br><br><u>Blunt vs. Penetrating, n(%)</u><br><br>N/A<br><br><u>TBI vs. multiple trauma, n(%)</u><br><br>39 (40.6) / 38 (45.2) vs. 57 (59.4) / 46 (54.8)	
Collaborators C-t, Shakur H, Roberts I, Bautista R, Caballero J, Coats T, et al. Effects of tranexamic acid on death, vascular occlusive events, and blood transfusion in trauma patients with significant haemorrhage (CRASH-2): a randomised, placebo-controlled trial. Lancet. 2010;376(9734):23-32.	**Region/Setting**<br><br>40 countries worldwide<br><br>May 2005 - March 2010<br><br>NCT00375258<br><br>**Inclusion criteria**<br><br>● significant haemorrhage (systolic blood pressure less than 90 mmHg and/or heart rate more than 110 beats per minute), or at risk of significant haemorrhage<br>● TXA administration within 8 hours of the injury<br>● ≥ 16 years old<br><br>**Exclusion criteria**<br><br>● Patients for whom the responsible doctor considers there is a clear indication for antifibrinolytic therapy<br>● Patients for whom there is considered to be a clear contraindication to antifibrinolytic therapy<br><br>**Patient characteristics IG/CG**<br><br><u>Age, years, mean (SD)</u><br><br>34.6 (14.1) / 34.5 (14.4)	**Intervention**<br><br>TXA bolus 1g, 30 min, maintenance 1g, 8h<br><br>**Control**<br><br>Placebo bolus, 10 min, maintenance, 8h<br><br>**Time of TXA administration after injury**<br><br>≤ 8 h<br><br>**Randomized patients**<br><br>10096/10115<br><br>**Follow-up**<br><br>28 days / hospital discharge

© 2022 Karl V et al. *JAMA Network Open*.

	Gender<br>Male 8439 (83.6%) 8496 (84.0%)<br>Vital signs<br>Shock defined as:<br>• Systolic blood pressure, mmHg<br>≤75: 1566 (15.55) / 1608 (15.9)<br>76-89: 1615 (16) / 1697 (16.8)<br>≥90: 6901 (68.4) / 6791 (67.1)<br>Not know: 11 (0.11) / 18 (0.18)<br>• Heart rate, bpm<br>≤77: 875 (8.7) / 871 (8.6)<br>77-91: 1727 (17.1) / 1770 (17.5)<br>92-107: 2556 (25.3) / 2546 (25.5)<br>>107: 4872 (48.3) / 4853 (48)<br>not know: 63 (0.62) / 74 (0.73)<br>If TBI: GCS score, points<br>3-8: 1799 (17.8) / 1839 (18.2)<br>9-12: 1353 (13.4) / 1351 (13.4)<br>13-15: 6934 (68.7) / 6908 (68.3)<br>Not known: 7 (0.07) / 16 (0.16)<br>Injury Severity Score, points, mean (SD)<br>N/A<br>Blunt vs. Penetrating, n (%)	

© 2022 Karl V et al. *JAMA Network Open*.

	6812 (67.5) / 6843 (67.7) vs. 3281 (32.5) / 3271 (32.3)<br><br>TBI vs. multiple trauma, n (%)<br><br>N/A	
Collaborators C-t. Effects of tranexamic acid on death, disability, vascular occlusive events and other morbidities in patients with acute traumatic brain injury (CRASH-3): a randomised, placebo-controlled trial. Lancet. 2019;394(10210):1713-23.	**Region/Setting**<br><br>29 countries worldwide<br><br>July 2012 - January 2019<br><br>NCT01402882<br><br>**Inclusion criteria**<br><br>Adults with traumatic brain injury who<br>● are within eight hours of injury (limited to within 3 hours from September, 2016)<br>● with any intracranial bleeding on CT scan or who have a GCS of 12 or less<br>● have no significant extracranial haemorrhage<br>● Responsible clinician's 'uncertainty' as to whether or not to use tranexamic acid in a particular patient with traumatic brain injury<br><br>**Exclusion criteria**<br><br>None<br><br>**Patient characteristics IG/CG**<br><br>Age, years, mean (SD)<br><br>41.7 (19.0) 41.9 (19.0)<br><br>Gender<br><br>Men 3742 (80) 3660 (80)<br><br>Women 906 (19) 893 (20)	**Intervention**<br><br>TXA bolus 1g, 30 min, maintenance 1g, 8h<br><br>**Control**<br><br>Placebo bolus, 10 min, maintenance, 8h<br><br>**Time of TXA administration after injury**<br><br>< 3 h<br><br>**Randomized patients**<br><br>4649 / 4553<br><br>**Follow-up**<br><br>28 days / hospital discharge / death

© 2022 Karl V et al. *JAMA Network Open*.

	<u>Vital signs</u><br>Shock defined as:<br>• Systolic blood pressure, mmHg<br><90: 89 (0.2) / 85 (2)<br>90-119: 1508 (32) / 1490 (33)<br>120-139: 1461 (31) / 1504<br>≥140: 1576 (34) / 1466 (32)<br>Not know: 15 (<1) / 8 (<1)<br>If TBI: GCS score, points, n (%)::<br>3: 495 (11) / 506 (11)<br>4: 213 (5) / 213 (5)<br>5: 163 (4) / 172 (4)<br>6: 221 (5) / 232 (5)<br>7: 311 (7) / 294 (6)<br>8: 354 (8) / 315 (7)<br>9: 335 (7) / 292 (6)<br>10: 371 (8) / 364 (8)<br>11: 375 (8) / 390 (9)<br>12: 476 (10) / 478 (10)<br>13: 297 (6) / 312 (7)<br>14: 526 (11) / 458 (10)<br>15: 484 (10) / 492 (11)<br>Unknown: 28 (1) / 35 (1)	

© 2022 Karl V et al. *JAMA Network Open*.

	Injury Severity Score, points, mean (SD)<br><br>N/A<br><br><br>Blunt vs. Penetrating, n (%)<br><br>N/A<br><br>TBI vs. multiple trauma, n (%)<br><br>N/A	
Guyette FX, Brown JB, Zenati MS, Early-Young BJ, Adams PW, Eastridge BJ, et al. Tranexamic Acid During Prehospital Transport in Patients at Risk for Hemorrhage After Injury: A Double-blind, Placebo-Controlled, Randomized Clinical Trial. JAMA Surgery. 2020;05:05.	**Region/Setting**<br><br>4 US locations<br><br>May 2015 - October 2019<br><br>NCT02086500<br><br>**Inclusion criteria**<br><br>● Blunt or penetrating injured patients at risk of bleeding being transported via air or ground medical services from the scene of injury or from referring hospital to a definitive trauma center that is participating in the trial AND<br>● Within 2 hours of time of injury AND<br>● Hypotension (Systolic Blood Pressure (SBP) < 90mmHg)<br>○ At scene of injury or during air or ground medical transport<br>○ Documented at referring hospital prior to air or ground medical transport arrival OR<br>● Tachycardia (heart rate >110 beats per minute)<br>○ At scene of injury or during air or ground medical transport<br>○ Documented at referring hospital prior to air or ground medical transport arrival<br><br>**Exclusion criteria**<br><br>● Age > 90 or < 18 years of age<br>● Inability to obtain intravenous access or intraosseous	**Intervention**<br><br>A) TXA bolus 1g, 10 min + Placebo bolus 10 min + Placebo infusion 8 h<br><br>B) TXA bolus 1g, 10 min + Placebo bolus 10 min + TXA infusion 8 h<br><br>C) TXA bolus 1g, 10 min + TXA bolus 10 min + TXA infusion 8 h<br><br>**Control**<br><br>Placebo bolus, 10 min + Placebo bolus 10 min + Placebo infusion 8 h<br><br>**Time of TXA administration after injury**<br><br>Prehospital<br><br>**Randomized patients**<br><br>460/467<br><br>**Follow-up**<br><br>30 days

© 2022 Karl V et al. *JAMA Network Open*.

	• Documented (radiographic evidence) cervical cord injury with motor deficit<br>• Known prisoner<br>• Known pregnancy<br>• Traumatic arrest with > 5 minutes CPR without return of vital signs<br>• Penetrating cranial injury<br>• Traumatic brain injury with brain matter exposed<br>• Isolated drowning or hanging victims<br>• Wearing an opt out bracelet<br>• Isolated fall from standing<br>• Patient or Family Objection at scene<br><br>**Patient characteristics IG/CG**<br><br><u>Age, years, mean (SD)</u><br><br>41 (17) / 42 (18)<br><br><u>Gender, n (%)</u><br><br>Male 327 (73.2) / 341 (74.8)<br><br><u>Vital signs</u><br><br>Shock defined as:<br><br>• Systolic blood pressure, mmHg, median (IQR) 123 (88-143) / 126 (87-148)<br>• Heart rate, bpm, median (IQR) 118 (112-127) / 117 (112-124)<br>If TBI: GCS score, n (%): GCS <8: 89 (19.9) / 107 (23.5)<br><br><u>Injury Severity Score, points, median (IQR)</u><br><br>13 (5-22) / 11 (4-22)<br><br><u>Blunt vs. Penetrating, n (%)</u><br><br>371 (83) / 389 (85.3) vs. 78 (17.4) / 70 (15.4)<br><br><u>TBI vs. multiple trauma, n (%)</u><br><br>N/A	

© 2022 Karl V et al. *JAMA Network Open*.

Rowell SE, Meier EN, McKnight B, Kannas D, May S, Sheehan K, et al. Effect of Out-of-Hospital Tranexamic Acid vs Placebo on 6-Month Functional Neurologic Outcomes in Patients With Moderate or Severe Traumatic Brain Injury. JAMA. 2020;324(10):961-74.	**Region/Setting**<br>20 US and Canada locations<br>May 2015 - November 2015<br>**Inclusion criteria**<br>● Blunt or penetrating traumatic mechanism consistent with traumatic brain injury<br>● Prehospital Glasgow Coma Score (GCS) score ≤ 12 at any time prior to randomization and administration of sedative and/or paralytic agents<br>● Prehospital systolic blood pressure (SBP) ≥ 90 mmHg prior to randomization<br>● Prehospital intravenous (IV) or intraosseous (IO) access<br>● Estimated Age ≥ 15 (or estimated weight > 50 kg if age is unknown)<br>● Emergency Medicine System (EMS) transport to a participating trauma center<br>**Exclusion criteria**<br>● Prehospital GCS=3 with no reactive pupil<br>● Estimated time from injury to hospital arrival > 2 hours<br>● Unknown time of injury - no known reference times to support estimation<br>● Clinical suspicion by EMS of seizure activity or known history of seizures, acute myocardial infarction (MI) or stroke<br>● Cardio-pulmonary resuscitation (CPR) by EMS prior to randomization<br>● Burns > 20% total body surface area (TBSA)<br>● Suspected or known prisoners<br>● Suspected or known pregnancy<br>● Prehospital TXA given prior to randomization<br>● Subjects who have activated the "opt-out" process when required by the local regulatory board<br>**Patient characteristics IG/CG**<br>Age, years, median (IQR)<br>A) 39 (26-57) B) 40 (26 / 56) / 36 (25-55)<br>Gender, n (%)	**Intervention**<br>A) 1g bolus TXA and 1g maintenance TXA, 8 h<br>B) 2 g bolus TXA, Placebo infusion<br>**Control**<br>Placebo bolus and maintenance<br>**Time of TXA administration after injury**<br>Prehospital<br>**Randomized patients**<br>718/345<br>**Follow-up**<br>6 months

© 2022 Karl V et al. *JAMA Network Open*.

	Male A) 227 (73) B) 255 (74) / 233 (75)<br><br><u>Vital signs</u><br><br>Shock defined as:<br><br>If TBI: GCS score, points, mean (SD)<br><br>7.8 (3.3) / 7.6 (3.2)<br><br><u>Injury Severity Score, points, median (IQR)</u><br><br>A) 17 (8-27) B) 17 (8-27) / 17 (9-27)<br><br><u>Blunt vs. Penetrating, n (%)</u><br><br>A) 302 (97) B) 339 (98) / 294 (95) vs. A) 12 (4) B) 5(1) / 16 (5)<br><br><u>TBI vs. multiple trauma, n (%)</u><br><br>N/A	
Yutthakasemsunt S, Kittiwatanagul W, Piyavechvirat P, Thinkamrop B, Phuenpathom N, Lumbiganon P. Tranexamic acid for patients with traumatic brain injury: a randomized, double-blinded, placebo-controlled trial. BMC Emergency Medicine. 2013;13:20.	**Region/Setting**<br><br>Thailand<br><br>October 2008 - October 2009<br><br>NCT00755209<br><br>**Inclusion criteria**<br><br>● moderate to severe injuries(post-resuscitation scores GCS 4-12)<br>● age ≥ 16 year<br>● non-penetrating TBI in 8 hours onset<br>● first CT brain in 8 hours onset<br>● non neurosurgical OR in 8 hours onset<br><br>**Exclusion criteria**<br><br>● patients with coagulopathy (only 1 excluded)<br>● serum creatinine over than 2 mg%<br>● with associated major organ injury requiring surgery within 8 hours of injury	**Intervention**<br><br>TXA<br><br>bolus 1g, 30 min<br><br>maintenance 1g, 8h<br><br>**Control**<br><br>Placebo<br><br>bolus, 10 min<br><br>maintenance, 8h<br><br>**Time of TXA administration after injury**<br><br>N/A<br><br>**Randomized patients**

© 2022 Karl V et al. *JAMA Network Open*.

	• pregnancy<br>• receiving any medication which affects haemostasis<br>**Patient characteristics IG/CG**<br>Age, years, mean (SD)<br>34.8 (16.0) / 34.1 (15.3)<br>Gender, n (%)<br>Male 103 (86%) / 107 (91%)<br>Vital signs<br>Shock defined as:<br>If TBI: GCS score, n (%): Moderate (9-12): 52 (53) / 47 (47); Severe (4-8): 68 (49) / 71 (51)<br>Injury Severity Score, points, mean (SD):<br>23.3 / 24.8<br>Blunt vs. Penetrating, n (%)<br>N/A<br>TBI vs. multiple trauma, n (%)<br>20 (17) / 16 (14) vs. 100 (83) / 102 (86)	120/120<br>**Follow-up**<br>hospital discharge
Bardes J, Palmer A, Con J, Wilson A & Schaefer G. Antifibrinolytics in a rural trauma state: Assessing the opportunities. Trauma Surgery and Acute Care Open. 2017; 0:1-4.	**Region/Setting**<br>USA<br>July 2013 - October 2014<br>**Inclusion criteria**<br>• Adult full trauma team activations (SBP <90mmHg, respiratory compromise, blood transfusions en route, patients requiring IV fluids to	**Intervention**<br>TXA 1 g bolus, 1 g maintenance<br>**Control**<br>No TXA<br>**Time of TXA administration after injury**

© 2022 Karl V et al. *JAMA Network Open*.

	maintain vital signs, penetrating trauma to neck or torso, or GCS <8 with traumatic mechanism)<br>● Indications for TXA: SBP <90mmHg, receiving blood transfusions, MTP activation or clinical concern for ongoing bleeding<br>**Exclusion criteria**<br>● Time since injury >3h<br>● Contraindications for TXA: >3h since injury, isolated TBI<br><br>**Patient characteristics IG/GC**<br>Age, years, mean (SD)<br>N/A<br>Gender<br>N/A<br>Vital signs<br>N/A<br>Injury Severity Score, points, mean (SD)<br>N/A<br>Blunt vs. Penetrating, n (%)<br>N/A<br>TBI vs. multiple trauma, n (%)<br>N/A	< 3 h<br>**Included Patients**<br>32 / 119<br>**Follow-up**<br>N/A
Boutonnet M, Abback P, Le Sache F, Harrois A, Follin A, Imbert N, et al. Tranexamic acid in severe trauma patients managed in a mature trauma care system. The Journal of Trauma and Acute Care	**Region/Setting**<br>Six French trauma centers<br>May 2011 - December 2015<br>**Inclusion criteria**	**Intervention**<br>TXA<br>**Control**<br>No TXA

© 2022 Karl V et al. *JAMA Network Open*.

Surgery. 2018;84(6S Suppl 1):S54-S62.	• > 16 years of age<br>• Major hemorrhage (defined by the need of four or more packed red blood cells [pRBC] in the first 6 hours following the trauma) or<br>• Received at least one pRBC in the emergency room (ER) or<br>• Received vasopressors either in the prehospital setting or in the ER<br><br>**Exclusion criteria**<br><br>None<br><br>**Patient characteristics IG/CG**<br><br>Age, years, mean (SD)<br><br>42 (19) (Propensity Score Calculation)<br><br>Gender, n (%)<br><br>Male 73.6 / 73.1 %<br><br>Vital signs<br><br>N/A<br><br>Injury Severity Score, points, n (%)<br><br><16: 143 (17.9%)<br><br>[16–30]: 322 (40.4%)<br><br>>30: 332 (41.7%)<br><br>(Propensity Score Calculation)<br><br>Blunt vs. Penetrating, n (%)<br><br>N/A<br><br>TBI vs. multiple trauma, n (%)<br><br>N/A	**Time of TXA administration after injury**<br><br>Prehospital or ER administration<br><br>**Included Patients**<br><br>403 / 281<br><br>**Follow-up**<br><br>90 days

© 2022 Karl V et al. *JAMA Network Open*.

Chan DYC, Tsang ACO, Li LF, Cheng KKF, Tsang FCP, Taw BBT, et al. Improving Survival with Tranexamic Acid in Cerebral Contusions or Traumatic Subarachnoid Hemorrhage: Univariate and Multivariate Analysis of Independent Factors Associated with Lower Mortality. World Neurosurgery. 2019;125:e665-e70.	**Region/Setting**<br><br>China, neurosurgery registry database<br>January 2010 - December 2016<br><br>**Inclusion criteria**<br><br>● Age >18 years<br>● Admission to the trauma center as an inpatient<br>● International Classification of Diseases (ICD) code of "Cerebral contusion (852.19)" or "Traumatic subarachnoid hemorrhage (852.00)" as the primary diagnosis<br><br>**Exclusion criteria**<br><br>● Other primary diagnosis ICD code such as "Diffuse axonal injury (850.4)," "Subdural hematoma, acute (852.20)," "Epidural hematoma (852.40)," "Concussion with no loss of consciousness (850.0)," or "Concussion with brief loss of consciousness (850.1)"<br>● Use of novel oral anticoagulants such as dabigatran, rivaroxaban, or apixaban<br><br>**Patient characteristics IG/CG**<br><br>Age, years, mean (SD)<br><br>66.4<br><br>Gender, n (%)<br><br>Male 410 (62.9)<br><br>Vital signs<br><br>If TBI: GCS score, points, mean (SD)<br><br>14-15: 473 (72.7)<br><br>9-13: 110 (16.9)	**Intervention**<br><br>1 g TXA on admission followed by 500 mg every 6 hours for 24 hours<br><br>**Control**<br><br>No TXA<br><br>**Time of TXA administration after injury**<br><br>On admission<br><br>**Included Patients**<br><br>81 / 570<br><br>**Follow-up**<br><br>N/A

© 2022 Karl V et al. *JAMA Network Open*.

	3-8: 68 (10.4)<br><br><u>Injury Severity Score, points, mean (SD)</u><br><br>N/A<br><br><u>Blunt vs. Penetrating, n (%)</u><br><br>N/A<br><br><u>TBI vs. multiple trauma, n (%)</u><br><br>N/A	
Cole E, Davenport R, Willett K, Brohi K. Tranexamic acid use in severely injured civilian patients and the effects on outcomes: a prospective cohort study. Annals of Surgery. 2015;261(2):390-4.	**Region/Setting**<br><br>England<br><br>**Inclusion criteria**<br><br>● > 15 years of age<br><br>**Exclusion criteria**<br><br>● ISS < 15<br><br>**Patient characteristics IG/CG**<br><br><u>Age, years, mean (SD)</u><br><br>42 (17.2) / 40 (18.6)<br><br><u>Gender, %</u><br><br>Male 78 / 82<br><br><u>Vital signs</u><br><br>Shock defined as:<br><br>• Systolic blood pressure, mmHg, mean (SD) 102 (34) / 127 (31)	**Intervention**<br><br>1g TXA bolus followed by 1g TXA infusion<br><br>**Control**<br><br>No TXA<br><br>**Time of TXA administration after injury**<br><br>Prehospital or ER, all < 3h<br><br>**Included Patients**<br><br>160 / 225<br><br>**Follow-up**<br><br>N/A

© 2022 Karl V et al. *JAMA Network Open*.

	• Base deficit, mEq/L, mean (SD) 7 (6) / 3 (5)<br><br><u>Injury Severity Score, points, mean (SD)</u><br><br>33 (13) / 29 (10)<br><br><u>Blunt vs. Penetrating, %</u><br><br>Blunt 84 / 93<br><br><u>TBI vs. multiple trauma, n (%)</u><br><br>TBI 65 / 64	
Dixon A, Emigh B, Spitz K, Teixeira P, Coopwood B, Trust M, et al. Does tranexamic acid really work in an urban US level I trauma center? A single level 1 trauma center's experience. American Journal of Surgery. 2019;218(6):1110-3.	**Region/Setting**<br><br>USA<br><br>2010 - 2017<br><br>**Inclusion criteria**<br><br>● Patients who underwent MTP defined as the receiving of greater than 10 units of pRBCs within the first 24 h of injury<br><br>**Exclusion criteria**<br><br>None<br><br>**Patient characteristics IG/CG**<br><br><u>Age, years, mean (SD)</u><br><br>41 (17) / 42 (17)<br><br><u>Gender, n (%)</u><br><br>Male 47 (76) / 43 (69)<br><br><u>Vital signs</u><br><br>Shock defined as:<br><br>• Systolic blood pressure, mmHg, mean (SD) 115 (32) / 98 (44)	**Intervention**<br><br>1g TXA bolus, 1 g TXA maintanance<br><br>**Control**<br><br>No TXA<br><br>**Time of TXA administration after injury**<br><br>Prehospital or ER<br><br>**Included Patients**<br><br>62 / 62<br><br>**Follow-up**<br><br>N/A

© 2022 Karl V et al. *JAMA Network Open*.

	• Heart rate, bpm, mean (SD) 103 (27) / 107 (34)<br><br>Injury Severity Score, points, mean (SD)<br><br>37 (13) / 36 (12)<br><br>Blunt vs. Penetrating, n (%)<br><br>Blunt 55 (89) / 49 (79)<br><br>TBI vs. multiple trauma, n (%)<br><br>N/A	
El-Menyar A, Sathian B, Wahlen BM, Abdelrahman H, Peralta R, Al-Thani H, et al. Prehospital administration of tranexamic acid in trauma patients: A 1:1 matched comparative study from a level 1 trauma center. American Journal of Emergency Medicine. 2020;38(2):266-71.	**Region/Setting**<br><br>Qatar<br><br>January 2017 - September 2018<br><br>**Inclusion criteria**<br><br>● Age 16 - 80<br>● Significant hemorrhage: SBP < 90mmHg and/or HR > 110 beats per minute OR at risk<br>● Within 3 h of trauma<br>● Control: No TXA administration but meeting CRASH-2 Criteria and receiving blood transfusion within the first 4 h<br>**Exclusion criteria**<br><br>● First dose of TXA at the ED<br>● < 16 and > 80 years of age<br>● Vulnerable population (prisoners and pregnant patients)<br>● Traumatic brain injury with exposed brain<br>● Isolated drowning or hanging victims<br><br>**Patient characteristics IG/CG**<br><br>Age, years, mean (95 % CI))<br><br>31.4 (29.2 - 33.7) / 31.5 (29.2 - 33.7)	**Intervention**<br><br>TXA 1 g<br><br>**Control**<br><br>No TXA<br><br>**Time of TXA administration after injury**<br><br>Prehospital<br><br>**Included Patients**<br><br>102 /102<br><br>**Follow-up**<br><br>N/A

© 2022 Karl V et al. *JAMA Network Open*.

	Gender, %<br><br>Male 96.1 / 89.2<br><br>Vital signs<br><br>Shock defined as:<br><br>• Systolic blood pressure, mmHg, mean (95 % CI) 106.9 (101.9 - 111.8) / 107.1 (101.9 - 112.3)<br>• Heart rate, bpm, mean (95 % CI) 97.1 (92.4 - 101.8) / 96.6 (90.9 - 102.2)<br>• Shock index, mean (SD) 0.96 (0.33) / 0.96 (0.34)<br>• Lactate, mmol/l, median (range) 3.0 (0.8 - 18.8) / 4.1 (0.6 - 18.0)<br>• Base value, mmol/l, median (range) 7.3 (-22.1 - 4.5) / -7.3 (-26.9 - 0.2)<br><br>Injury Severity Score, points, mean (95 % CI)<br><br>24.0 (21.4 - 26.5) / 25 (22.3 - 27.6)<br><br>Blunt vs. Penetrating, n (%)<br><br>N/A<br><br>TBI vs. multiple trauma, n (%)<br><br>N/A	
Glover TE, Sumpter JE, Ercole A, Newcombe VFJ, Lavinio A, Carrothers AD, et al. Pulmonary embolism following complex trauma: UK MTC observational study. Emergency Medicine Journal. 2019;36(10):608-12.	**Region/Setting**<br><br>UK<br><br>November 2014 - May 2017<br><br>**Inclusion criteria**<br><br>● Age 18 years and older<br>● Major trauma (undefined)<br>● Admitted to a level 2 or 3 trauma center<br><br>**Exclusion criteria**<br><br>N/A	**Intervention**<br><br>TXA<br><br>**Control**<br><br>No TXA<br><br>**Time of TXA administration after injury**<br><br>N/A<br><br>**Included Patients**

© 2022 Karl V et al. *JAMA Network Open*.

	**Patient characteristics IG/CG**<br><br><u>Age, years, median (range)</u><br><br>45.3 (18 - 95.7) / 51.9 (18 - 96.1)<br><br><u>Gender, %</u><br><br>Male 75.7 / 72.1<br><br><u>Vital signs</u><br><br>N/A<br><br><u>Injury Severity Score, points, median (range)</u><br><br>27 (4 - 75) / 25 (4 - 59)<br><br><u>Blunt vs. Penetrating, n (%)</u><br><br>N/A<br><br><u>TBI vs. multiple trauma, n (%)</u><br><br>N/A	333 / 706<br><br>**Follow-up**<br><br>6 weeks
Harvin JA, Peirce CA, Mims MM, Hudson JA, Podbielski JM, Wade CE, et al. The impact of tranexamic acid on mortality in injured patients with hyperfibrinolysis. The Journal of Trauma and Acute Care Surgery. 2015;78(5):905-9; discussion 9-11.	**Region/Setting**<br><br>USA<br><br>September 2009 - September 2013<br><br>**Inclusion criteria**<br><br>● Age 16 years and older<br>● Evidence of hyperfibrinolysis (rTEG LY-30 of 3 % or greater)<br><br>**Exclusion criteria**<br><br>● Burn wounds > 20 % of total body surface<br>● Dead within 30 minutes of arrival	**Intervention**<br><br>TXA 1 g bolus, 1 g infusion over 8 h<br><br>was implemented in 2011 (study started 2009) where 1g bolus and 1g maintenance dose of TXA was administered —> 2 years without a guideline and missing of dose regime<br><br>**Control**<br><br>No TXA<br><br>**Time of TXA administration after injury**

© 2022 Karl V et al. *JAMA Network Open*.

	**Patient characteristics IG/CG**<br><br>Age, years, median (IQR)<br><br>37 (26 - 56) / 32 (23 - 46)<br><br>Gender, %<br><br>Male 80 / 74<br><br>Vital signs<br><br>Shock defined as:<br><br>• Systolic blood pressure, mmHg, median (IQR) 121 (100 - 140) / 126 (104 / 143)<br>• Heart rate, bpm, median (IQR) 105 (84 - 125) / 100 (84 - 120)<br>• Lactate, mmol/l, median (IQR) 4.4 (3.1 - 7.7) / 3 (1.9 - 4.6)<br>• Base value, mmol/l, median (IQR) -5 (-12 - -2) / -2 (-5 - 1)<br><br>Injury Severity Score, points, mean (SD)<br><br>29 (19 - 41) / 14 (4 - 25)<br><br>Blunt vs. Penetrating, n (%)<br><br>Blunt 78 / 65<br><br>TBI vs. multiple trauma, n (%)<br><br>N/A	< 3 h<br><br>**Included Patients**<br><br>98 / 934<br><br>**Follow-up**<br><br>N/A
Howard JT, Stockinger ZT, Cap AP, Bailey JA, Gross KR. Military use of tranexamic acid in combat trauma: Does it matter? The Journal of Trauma and Acute Care Surgery. 2017;83(4):579-88.	**Region/Setting**<br><br>USA<br><br>October 2010 - March 2014<br><br>**Inclusion criteria**<br><br>● Injured in combat in Afghanistan<br>● Been admitted to role 3 MTF	**Intervention**<br><br>TXA<br><br>**Control**<br><br>No TXA

© 2022 Karl V et al. *JAMA Network Open*.

	• Received blood transfusion of at least one unit<br><br>**Exclusion criteria**<br><br>None<br><br>**Patient characteristics IG/CG**<br><br>Age, years, mean (SD)<br><br>24.6 (9) / 24.9 (10.1)<br><br>Gender, n (%)<br><br>Male 362 (97.1) / 627 (95.4)<br><br>Vital signs<br><br>N/A<br><br>Injury Severity Score, points, mean (SD)<br><br>16.8 (10.6) / 16.2 (10.2)<br><br>Blunt vs. Penetrating, n (%)<br><br>N/A<br><br>TBI vs. multiple trauma, n (%)<br><br>N/A	**Time of TXA administration after injury**<br><br>N/A<br><br>**Included Patients**<br><br>373 / 657<br><br>**Follow-up**<br><br>Discharge / 30 days
Johnston LR, Rodriguez CJ, Elster EA, Bradley MJ. Evaluation of Military Use of Tranexamic Acid and Associated Thromboembolic Events. JAMA Surgery. 2018;153(2):169-75.	**Region/Setting**<br><br>USA<br><br>2011-2015<br><br>**Inclusion criteria**<br><br>Treated at the Walter Reed National Military Medical Center<br><br>**Exclusion criteria**	**Intervention**<br><br>TXA<br><br>**Control**<br><br>None<br><br>**Time of TXA administration after injury**<br><br>N/A

© 2022 Karl V et al. *JAMA Network Open*.

	N/A<br><br>**Patient characteristics IG/CG**<br><br>Age, years, mean (SD)<br><br>25.3 (4.8) / 27.4 (7.1)<br><br>Gender, n (%)<br><br>Male 144 (99.3) / 299 (96.4)<br><br>Vital signs<br><br>Shock defined as:<br><br>• Systolic blood pressure, mmHg, mean (SD) 115.5 (30.8) / 131.4 (23.9)<br>• Heart rate, bpm, mean (SD) 120.6 (30) / 92.8 (4.4)<br><br>**Injury Severity Score, points, mean (SD)**<br><br>27.8 (12.4) / 15.6 (10.8)<br><br>Blunt vs. Penetrating, n (%)<br><br>N/A<br><br>TBI vs. multiple trauma, n (%)<br><br>N/A	**Included Patients**<br><br>146 / 309<br><br>**Follow-up**<br><br>through initial hospitalization and readmission
Khan M, Jehan F, Bulger EM, O'Keeffe T, Holcomb JB, Wade CE, et al. Severely injured trauma patients with admission hyperfibrinolysis: Is there a role of tranexamic acid? Findings from the PROPPR trial. The Journal of Trauma and Acute Care Surgery. 2018;85(5):851-7.	**Region/Setting**<br><br>USA<br><br>**Inclusion criteria**<br><br>● Adult trauma patients<br>● Hyperfibrinolysis, defined as Ly30 >/= 3 %<br><br>**Exclusion criteria**<br><br>● Received TXA > 3h	**Intervention**<br><br>TXA<br><br>**Control**<br><br>No TXA<br><br>**Time of TXA administration after injury**<br><br>< 3h

© 2022 Karl V et al. *JAMA Network Open*.

	**Patient characteristics IG/CG**<br><br><u>Age, years, mean (SD)</u><br><br>42.5 (20) / 38.7 (17)<br><br><u>Gender, %</u><br><br>Male 66 / 68<br><br><u>Vital signs</u><br><br>Shock defined as:<br><br>• Systolic blood pressure, mmHg, median (IQR) 100 (76 - 127) / 101 (80 - 131)<br>• Heart rate, bpm, median (IQR) 115 (76 - 127) / 120 (100 - 134)<br>• Lactate, mmol/l, median (IQR) 8.3 (5.1 - 11.7) / 9.5 (5.1 - 12.7)<br>• Base value, mmol/l, median (IQR) -14 (-18 - -8) / -12 (-18 - -6.7)<br><br><u>Injury Severity Score, points, median (IQR)</u><br><br>38 (23 -45) / 35 (21 - 45)<br><br><u>Blunt vs. Penetrating, %</u><br><br>Penetrating 32 / 44<br><br><u>TBI vs. multiple trauma, n (%)</u><br><br>N/A	**Included Patients**<br><br>31 / 62<br><br>**Follow-up**<br><br>death/ discharge / 30 days after admission
Lewis CJ, Li P, Stewart L, Weintrob AC, Carson ML, Murray CK, et al. Tranexamic acid in life-threatening military injury and the associated risk of infective complications. British Journal of Surgery. 2016;103(4):366-73.	**Region/Setting**<br><br>USA<br><br>June 2009 - December 2013<br><br>**Inclusion criteria**<br><br>● received blood products (i.e. packed red blood cells, whole blood) within 24 h of injury<br>**Exclusion criteria**	**Intervention**<br><br>TXA<br><br>**Control**<br><br>No TXA<br><br>**Time of TXA administration after injury**

© 2022 Karl V et al. *JAMA Network Open*.

	N/A<br>**Patient characteristics IG/CG**<br>Age, years, median (IQR)<br>24.2 (21.7 - 27.2) / 24.2 (21.8 - 28.7)<br>Gender, n (%)<br>Male 334 (90.7) / 570 (91.1)<br>Vital signs<br>Shock defined as:<br>• Shock index, median (IQR) 1.1 (0.8 - 1.4) / 0.9 (0.7 - 1.2)<br>Injury Severity Score, points, median (IQR)<br>33 (27 - 45) / 33 (26 - 43)	N/A<br>**Included Patients**<br>335 / 626<br>**Follow-up**<br>N/A
Luehr E, Grone G, Pathak M, Austin C, Thompson S. Administration of tranexamic acid in trauma patients under stricter inclusion criteria increases the treatment window for stabilization from 24 to 48 hours-a retrospective review. International Journal of Burns & Trauma. 2017;7(6):115-9.	**Region/Setting**<br>USA<br>2013 - 2016<br>**Inclusion criteria**<br>● age 16 and older<br>● no known hypertensivity to TXA<br>● no known severe renal failure<br>● no known history of thromboembolism<br>● patient does not present with aneurismal subarachnoid hemorrhage<br>● patient is seen by qualified medical personnel within 3 h of injury<br>● survived ≥ 8.5 hrs<br>● received at least a single blood product<br>● BPM ≥ 120 and SBP ≤ 90 mmHg<br>**Exclusion criteria**	**Intervention**<br>TXA<br>**Control**<br>No TXA<br>**Time of TXA administration after injury**<br>< 3h<br>**Included Patients**<br>53 / 62<br>**Follow-up**<br>30 days

© 2022 Karl V et al. *JAMA Network Open*.

	- Exclusion of isolated injuries, such as orthopedic, face and neurosurgical patients. **Patient characteristics IG/CG**<br>Age, years, mean (SD)<br>41.6 (18.3) / 41.9 (18.6)<br>Gender,n %<br>42 (79.3) / 36 (58.1)<br>Vital signs<br>Shock defined as: - Systolic blood pressure, mmHg, mean (SD) 77.3 (10) / 77 (10.6) - Heart rate, bpm, mean (SD) 131.5 (8.2) / 135.5 (13) Injury Severity Score, points, mean (SD)<br>21.6 (11.4) / 20.3 (11.7)<br>Blunt vs. Penetrating, n (%)<br>Blunt 43 (81.1) / 53 (85.5)<br>TBI vs. multiple trauma, n (%)<br>N/A	
Moore HB, Moore EE, Huebner BR, Stettler GR, Nunns GR, Einersen PM, et al. Tranexamic acid is associated with increased mortality in patients with physiological fibrinolysis. Journal of Surgical Research. 2017;220:438-43.	**Region/Setting**<br>USA<br>2014 - 2016<br>**Inclusion criteria** NISS > 15 **Exclusion criteria**	**Intervention**<br>TXA<br>**Control**<br>No TXA<br>**Time of TXA administration after injury**

© 2022 Karl V et al. *JAMA Network Open*.

	N/A<br>**Patient characteristics IG/CG**<br>Age, years, median (interquartile values)<br>27 (24 - 54) / 34 (27 - 49)<br>Gender, %<br>Male 85 / 77<br>Vital signs<br>N/A<br>New Injury Severity Score, points, median (interquartile values)<br>48 (29 - 57) / 29 (22 - 43)<br>Blunt vs. Penetrating, n (%)<br>Penetrating 42 / 32<br>TBI vs. multiple trauma, n (%)<br>N/A	N/A<br>**Included Patients**<br>26 / 206<br>**Follow-up**<br>Discharge/death
Morrison JJ, Dubose JJ, Rasmussen TE, Midwinter MJ. Military Application of Tranexamic Acid in Trauma Emergency Resuscitation (MATTERs) Study. Archives of Surgery. 2012;147(2):113-9.	**Region/Setting**<br>UK<br>January 2009 - December 2010<br>**Inclusion criteria**<br>• Massive transfusion (at least 10 units of packed red blood cells)<br>**Exclusion criteria**<br>N/A<br>**Patient characteristics IG/CG**	**Intervention**<br>TXA bolus of 1 g, repeated as felt indicated by the managing clinician<br>**Control**<br>No TXA<br>**Time of TXA administration after injury**<br>N/A<br>**Included Patients**

© 2022 Karl V et al. *JAMA Network Open*.

	<u>Age, years, mean (SD)</u><br>23.8 (7.7) / 22.9 (9.2)<br><u>Gender, %</u><br>Male 98.4 / 96.9<br><u>Vital signs</u><br>N/A<br><u>Injury Severity Score, points, mean (SD)</u><br>26.1 (17.1) / 25.2 (20.5)<br><u>Blunt vs. Penetrating, n (%)</u><br>N/A<br><u>TBI vs. multiple trauma, n (%)</u><br>N/A	125 / 196<br>**Follow-up**<br>discharge/ 30 days
Morrison JJ, Ross JD, Dubose JJ, Jansen JO, Midwinter MJ, Rasmussen TE. Association of cryoprecipitate and tranexamic acid with improved survival following wartime injury: findings from the MATTERs II Study. JAMA Surgery. 2013;148(3):218-25.	**Region/Setting**<br>UK<br>March 2006 - March 2011<br>**Inclusion criteria**<br>• patients who received at least 1 unit of packed red blood cells<br>**Exclusion criteria**<br>N/A<br>**Patient characteristics IG/CG**<br><u>Age, years, mean (SD)</u><br>24.2 (11.7) / 23.6 (1.6)	**Intervention**<br>TXA bolus of 1 g intravenous, further doses at the clinician's discretion.<br>**Control**<br>No TXA<br>**Time of TXA administration after injury**<br>N/A<br>**Included Patients**<br>148 / 758<br>**Follow-up**

© 2022 Karl V et al. *JAMA Network Open*.

	Gender, n (%)<br>Male 143 (96.6) / 710 (93.7)<br>Vital signs<br>N/A<br>Injury Severity Score, points, mean (SD)<br>23 (19.2) / 21.2 (18.5)<br>Blunt vs. Penetrating, n (%)<br>N/A<br>TBI vs. multiple trauma, n (%)<br>N/A	discharge / 30 days
Morte D, Lammers D, Bingham J, Kuckelman J, Eckert M, Martin M. Tranexamic acid administration following head trauma in a combat setting: Does tranexamic acid result in improved neurologic outcomes? The Journal of Trauma and Acute Care Surgery. 2019;87(1):125-9.	**Region/Setting**<br>USA<br>2008 - 2015<br>**Inclusion criteria**<br>● Adult trauma patients<br>● Documented Head Abbreviated Injury Scale<br>● Requiring blood product resuscitation or<br>● Likely to require massive transfusion (greater than 10 units of BP over 24 h)<br>**Exclusion criteria**<br>● No documented Head Abbreviated Injury Scale<br>● Incomplete data<br>**Patient characteristics IG/CG**<br>Age, years, mean	**Intervention**<br>1 g TXA bolus, 1 g TXA maintenance<br>**Control**<br>No TXA<br>**Time of TXA administration after injury**<br>N/A<br>**Included Patients**<br>46 / 46<br>**Follow-up**<br>discharge

© 2022 Karl V et al. *JAMA Network Open*.

	24.7 / 25.3<br>Gender, n (%)<br>Male 46 (100) / 46 (100)<br>Vital signs<br>GCS score, points, n (%)<br>GCS 14-15: 23 (50) / 21 (45.7)<br>GCS 10-13: 5 (10.9) / 3 (6.5)<br>GCS <9: 18 (39.1) / 22 (47.8)<br>Injury Severity Score, points, mean (SD)<br>25.1 / 25.7<br>Blunt vs. Penetrating, n (%)<br>Penetrating 43 (93.5) / 39 (82.6)<br>TBI vs. multiple trauma, n (%)<br>N/A	
Myers SP, Kutcher ME, Rosengart MR, Sperry JL, Peitzman AB, Brown JB, et al. Tranexamic acid administration is associated with an increased risk of posttraumatic venous thromboembolism. The Journal of Trauma and Acute Care Surgery. 2019;86(1):20-7.	**Region/Setting**<br>USA<br>January 2012 - December 2016<br>**Inclusion criteria**<br>• Adult trauma patients<br>**Exclusion criteria**<br>• taking prehospital anticoagulation<br>• known risk of DVT, PE, or hereditary coagulopathy<br>• prehospital TXA administration as part of a separate trial protocol	**Intervention**<br>TXA<br>**Control**<br>No TXA<br>**Time of TXA administration after injury**<br>N/A<br>**Included Patients**<br>189 / 189

© 2022 Karl V et al. *JAMA Network Open*.

	**Patient characteristics IG/CG**<br><br>Age, years, median (IQR)<br><br>36 (24 - 53) / 32 (22 - 55)<br><br>Gender, n (%)<br><br>Male 142 (75) / 132 (70)<br><br>Vital signs<br><br>Shock defined as:<br><br>• Systolic blood pressure, mmHg, median (IQR) 83 (70, 98) / 84 (71, 98)<br>• Heart rate, bpm, median (IQR) 130 (114, 141) / 119 (104, 132)<br>• Lactate, mmol/l, mean (SD) 6.0 (3.8) / 5.4 (4.8)<br><br>Injury Severity Score, points, median (IQR)<br><br>19 (12, 27) / 14 (8, 22)<br><br>Blunt vs. Penetrating, n (%)<br><br>Penetrating 65 (44) / 40 (21)<br><br>TBI vs. multiple trauma, n (%)<br><br>N/A	**Follow-up**<br><br>Discharge
Neeki MM, Dong F, Toy J, Vaezazizi R, Powell J, Wong D, et al. Tranexamic Acid in Civilian Trauma Care in the California Prehospital Antifibrinolytic Therapy Study. The Western Journal of Emergency Medicine. 2018;19(6):977-86.	**Region/Setting**<br><br>USA<br><br>March 2015 - July 2017<br><br>**Inclusion criteria**<br><br>● Blunt or penetrating trauma with signs and symptoms of hemorrhagic shock within three hours of injury (SBP less than 90 mmHg at scene of injury, during air and/or ground medical transport, or upon arrival to designated trauma centers; Heart rate > 120; Estimated blood loss of 500 milliliters in the field.	**Intervention**<br><br>1 g TXA bolus, 1 g TXA maintenance<br><br>**Control**<br><br>No TXA<br><br>**Time of TXA administration after injury**<br><br><3 h

© 2022 Karl V et al. *JAMA Network Open*.

	• Bleeding not controlled by direct pressure or tourniquet<br>• Major amputation of any extremity above the wrists and above the ankles<br><br>**Exclusion criteria**<br><br>• Any patient <18 years of age<br>• Any patient more than three hours post-injury<br>• Any patient with an active thromboembolic event (within the last 24 hours) – i.e., active stroke, myocardial infarction or pulmonary embolism<br>• Any patient with a hypersensitivity or anaphylactic reaction to TXA<br>• Traumatic arrest with more than five minutes of cardiopulmonary resuscitation without return of vital signs<br>• Penetrating cranial injury<br>• Traumatic brain injury with brain matter exposed<br>• Isolated drowning or hanging victims<br>• Documented cervical cord injury with motor deficits<br><br>**Patient characteristics IG/CG**<br><br>Age, years, mean (SD)<br><br>37.96 (16.11) / 37.64 (16.33)<br><br>Gender,n (%)<br><br>Male 293 (80.9) / 293 (80.9)<br><br>Vital signs<br><br>Shock defined as:<br><br>• Systolic blood pressure, mmHg, mean (SD) 78.42 (16.17) / 83.66 (14.13)<br><br>Injury Severity Score, points, mean (SD)<br><br>16.08 (10.69) / 17.15 (11.71)<br><br>Blunt vs. Penetrating, n (%)	**Included Patients**<br><br>362 / 362<br><br>**Follow-up**<br><br>28 days

© 2022 Karl V et al. *JAMA Network Open*.

	Penetrating 228 (63) / 228 (63)<br><br><u>TBI vs. multiple trauma, n (%)</u><br><br>N/A	
Neeki MM, Dong F, Toy J, Salameh J, Rabiei M, Powell J, et al. Safety and Efficacy of Hospital Utilization of Tranexamic Acid in Civilian Adult Trauma Resuscitation. The Western Journal of Emergency Medicine. 2020;21(2):217-25.	**Region/Setting**<br><br>USA<br><br>June 2014 - July 2018<br><br>**Inclusion criteria**<br><br>● Blunt or penetrating trauma with signs and symptoms of hemorrhagic shock within three hours of injury (SBP of less than 90 mmHg upon arrival to designated trauma centers, HR > 120, estimated blood loss of 500 milliliters, bleeding not controlled by direct pressure or tourniquet<br>● Major amputation of any extremity above the wrists and above the ankles<br><br>**Exclusion criteria**<br><br>● Any patient <18 years of age<br>● Any patient more than three hours post-injury<br>● Any patient with an active thromboembolic event (within the last 24 hours) – i.e., active stroke, myocardial infarction or pulmonary embolism<br>● Any patient with a hypersensitivity or anaphylactic reaction to TXA<br>● Any patient that received prehospital TXA<br>● Traumatic arrest with more than five minutes of cardiopulmonary resuscitation without return of vital signs<br>● Penetrating cranial injury<br>● Traumatic brain injury with brain matter exposed<br>● Isolated drowning or hanging victims<br>● Documented cervical cord injury with motor deficits<br><br>**Patient characteristics IG/CG**<br><br><u>Age, years, mean (SD)</u>	**Intervention**<br><br>TXA 1 g bolus, 1 g maintenance<br><br>**Control**<br><br>No TXA<br><br>**Time of TXA administration after injury**<br><br><3 h<br><br>**Included Patients**<br><br>280 / 280<br><br>**Follow-up**<br><br>28 days

© 2022 Karl V et al. *JAMA Network Open*.

	38.89 (15.98) / 37.91 (18.15)<br><br><u>Gender, n (%)</u><br><br>Male 236 (84.3) / 241 (86.1)<br><br><u>Vital signs</u><br><br>Shock defined as:<br><br>Systolic blood pressure, mmHg, mean (SD) 99.32 (17.84) / 102.32 (23.27)<br><br><u>Injury Severity Score, points, mean (SD)</u><br><br>N/A<br><br><u>Blunt vs. Penetrating, n (%)</u><br><br>Blunt 159 (56.8) / 160 (57.1)<br><br><u>TBI vs. multiple trauma, n (%)</u><br><br>N/A	
Shiraishi A, Kushimoto S, Otomo Y, Matsui H, Hagiwara A, Murata K, et al. Effectiveness of early administration of tranexamic acid in patients with severe trauma. British Journal of Surgery. 2017;104(6):710-7.	**Region/Setting**<br><br>Japan<br><br>2012<br><br>**Inclusion criteria**<br><br>● 18 years of age<br>● ISS ≥ 16<br><br>**Exclusion criteria**<br><br>● Complications (e.g. out-of-hospital cardiac arrest, burns, liver cirrhosis, isolated cervical spine injury not caused by a high-energy accident)<br>● Pregnancy<br><br>**Patient characteristics IG/CG**	**Intervention**<br><br>TXA<br><br>**Control**<br><br>No TXA<br><br>**Time of TXA administration after injury**<br><br><3 h<br><br>**Included Patients**<br><br>250 / 250<br><br>**Follow-up**<br><br>28 days

© 2022 Karl V et al. *JAMA Network Open*.

	<u>Age, years, median (IQR)</u><br>57 (36 - 72) / 56 (38 - 69)<br><u>Gender, n (%)</u><br>181 (72.4) / 186 (74.4)<br><u>Vital signs</u><br>Shock defined as:<br>• Systolic blood pressure, mmHg, median (IQR) 136 (110 - 159) / 133 (110 - 157)<br>• Heart rate, bpm, median (IQR) 87 (75 - 100) / 86 (75 - 100)<br>• Lactate, mmol/l, mean (SD) 2.6 (1.7 - 4) / 2.6 (1.6 - 3.9)<br><u>Injury Severity Score, points, median (IQR)</u><br>25 (17-29) / 25 (17-29)<br><u>Blunt vs. Penetrating, n (%)</u><br>Blunt 249 (99.6) / 249 (99.6)<br><u>TBI vs. multiple trauma, n (%)</u><br>TBI 188 (75.2) / 190 (76)	
Swendsen H, Galante J, Utter G, Bateni S, Scherer L, Schermer C. Tranexamic Acid use in Trauma: Effective but not Without Consequences. 2013.	**Region/Setting**<br>USA<br>December 2011 - July 2012<br>**Inclusion criteria**<br>● age 18 years and older<br>● Hypotension (SBP <90 mm Hg) upon presentation<br>● Massive transfusion guideline activation in the Emergency<br>● Department (ED)<br>● Transport directly to the operating room (OR) or interventional radiology (IR) suite from the ED	**Intervention**<br>TXA 1 g bolus, 1 g maintenance<br>**Control**<br>No TXA<br>**Time of TXA administration after injury**<br>< 3 h<br>**Included Patients**

© 2022 Karl V et al. *JAMA Network Open*.

	**Exclusion criteria**<br>● patients who were transferred from another hospital<br>● injured more than 3 hours prior the TXA administration<br>**Patient characteristics IG/CG**<br>Age, years, mean (SD)<br>44.6 (20.3) / 47.6 (18.9)<br>Gender, %<br>37 (71.2) / 49 (66.2)<br>Vital signs<br>Shock defined as:<br>• Systolic blood pressure, mmHg, mean (SD) 83 (26) / 74 (20)<br>• Heart rate, bpm, mean (SD) 127.4 (25.5) / 117 (25)<br>Injury Severity Score, points, mean (SD)<br>27.1 (15) / 20.5 (16.8)<br>Blunt vs. Penetrating, n (%)<br>N/A<br>TBI vs. multiple trauma, n (%)<br>N/A	52 / 74<br>**Follow-up**<br>Discharge
Valle EJ, Allen CJ, Van Haren RM, Jouria JM, Li H, Livingstone AS, et al. Do all trauma patients benefit from tranexamic acid? The Journal of Trauma and Acute Care Surgery. 2014;76(6):1373-8.	**Region/Setting**<br>USA<br>August 2009 - January 2013<br>**Inclusion criteria**<br>● Adult trauma patients	**Intervention**<br>TXA 1 g bolus, 1 g maintenance<br>**Control**<br>No TXA

© 2022 Karl V et al. *JAMA Network Open*.

	• Underwent emergency surgery directly from the resuscitation area<br><br>**Exclusion criteria**<br><br>• Emergency surgery for isolated orthopedic and/or neurosurgical indications and minor trauma operations such as those for complex wound closures<br><br>**Patient characteristics IG/CG**<br><br><u>Age, years, mean (SD)</u><br><br>42 (20) / 43 (20)<br><br><u>Gender, %</u><br><br>Male 85 / 86<br><br><u>Vital signs</u><br><br>Shock defined as:<br><br>• Systolic blood pressure, mmHg, mean (SD) 98 (30) / 101 (32)<br>• Heart rate, bpm, mean (SD) 105 (26) / 103 (29)<br>• Base value, mmol/l, mean (SD) -7.4 (7) / -7.7 (6.9)<br><br><u>Injury Severity Score, points, mean (SD)</u><br><br>28 (16) / 28 (17)<br><br><u>Blunt vs. Penetrating, %</u><br><br>Penetrating 54 / 54<br><br><u>TBI vs. multiple trauma, n (%)</u><br><br>TBI 24 / 26	**Time of TXA administration after injury**<br><br>N/A<br><br>**Included Patients**<br><br>150 / 150<br><br>**Follow-up**<br><br>N/A

© 2022 Karl V et al. *JAMA Network Open*.

Wafaisade A, Lefering R, Bouillon B, Bohmer AB, Gasler M, Ruppert M, et al. Prehospital administration of tranexamic acid in trauma patients. Critical Care (London, England). 2016;20(1):143.	**Region/Setting**<br><br>Germany<br><br>January 2012 - December 2014<br><br>**Inclusion criteria**<br><br>1. ADAC Air Rescue Service database:<br><br>● Primarily admitted trauma patient<br>● Critical injury, defined as preclinically assessed NACA IV (potentially life-threatening), NACA V (acute danger) or NACA VI (respiratory and/or cardiac arrest)<br>● Admission to a trauma center participating in the TR-DGU<br>2. TR-DGU database:<br><br>● Primary admission<br>● Treatment in a German trauma centre (i.e., exclusion of trauma centres from other countries)<br><br>**Exclusion criteria**<br><br>N/A<br><br>**Patient characteristics IG/CG**<br><br><u>Age, years, mean (SD)</u><br><br>43 (19) / 41 (18)<br><br><u>Gender, n (%)</u><br><br>Male 187 (72.5) / 187 (72.5)<br><br><u>Vital signs</u><br><br>Shock defined as:<br><br>• Systolic blood pressure, mmHg, mean (SD) 118 (34) / 116 (33)<br>• Base value, mmol/l, mean (SD) -3.1 (4.7) / -3.5 (5.1)	**Intervention**<br><br>TXA<br><br>**Control**<br><br>No TXA<br><br>**Time of TXA administration after injury**<br><br><3 h<br><br>**Included Patients**<br><br>258 / 258<br><br>**Follow-up**<br><br>30 days

© 2022 Karl V et al. *JAMA Network Open*.

	Injury Severity Score, points, mean (SD)<br><br>24 (14) / 24 (16)<br><br>Blunt vs. Penetrating, n (%)<br><br>Blunt 233 (90.3) / 240 (93)<br><br>TBI vs. multiple trauma, n (%)<br><br>Isolated TBI 7 (2.7) / 8 (3.1)	
Walker PF, Bozzay JD, Johnston LR, Elster EA, Rodriguez CJ, Bradley MJ. Outcomes of tranexamic acid administration in military trauma patients with intracranial hemorrhage: a cohort study. BMC Emergency Medicine. 2020;20(1):39.	**Region/Setting**<br><br>USA<br><br>October 2010 - December 2015<br><br>**Inclusion criteria**<br><br>● military casualties<br>● diagnosed with military combat-related ICH<br><br>**Exclusion criteria**<br><br>● no active duties service members<br>● incomplete data<br><br>**Patient characteristics IG/CG**<br><br>Age, years, mean (SD)<br><br>24.2 (3.1) / 25.5 (5.3)<br><br>Gender, %<br><br>N/A<br><br>Vital signs<br><br>If TBI: GCS score, points, mean (SD) 9.2 (4.4) / 12.5 (3.4)<br><br>Injury Severity Score, points, mean (SD)	**Intervention**<br><br>TXA<br><br>**Control**<br><br>No TXA<br><br>**Time of TXA administration after injury**<br><br>N/A<br><br>**Included Patients**<br><br>14 / 57<br><br>**Follow-up**<br><br>N/A

© 2022 Karl V et al. *JAMA Network Open*.

	36.6 (12.5) / 26.2 (11.4)<br><br>Blunt vs. Penetrating, n (%)<br><br>Penetrating TBI 6 (42.3) / 30 (52.6)<br><br>TBI vs. multiple trauma, n (%)<br><br>N/A	

Abbreviations: IG: Intervention group; CG: Control group; SD: Standard deviation; GCS: Glasgow Coma Score; TXA: Tranexamic acid; ED: Emergency department; TBI: Traumatic brain injury; N/A: not available; SBP: Systolic blood pressure; HR: Heart rate; ICH: Intracranial hemorrhage

© 2022 Karl V et al. *JAMA Network Open*.

eTable 3. **Extracted Data of Blood Transfusions**

study	unit.e	sd.e	unit.c	sd.c	PLT mean.e	PLT sd.e	PLT mean.c	PLT sd.c	WB mean.e	WB sd.e	WB mean.c	WB sd.c	PRBC mean.e	PRBC sd.e	PRBC mean.c	PRBC sd.c	Cryo mean.e	Cryo sd.e	Cryo mean.c	Cryo sd.c	FFP mean.e	FFP sd.e	FFP mean.c	FFP sd.c	Follow-up
Chakroun-Walha 2019 RCT	1.6		0.5		0.3	1.4	0	0					0.5	1.2	0.2	0.6					0.8	1.0	0.3	1.6	24 h
CRASH-2 RCT	3		3																						D/28 d
Guyette 2020 RCT	0		0		0		0						0		0						0		0		24h
Balvers 2017	25	28	20	17																					24 h
Cole 2015					1	1	0	0.7					7	7.4	2	5.0	1	1	0	0.1	5	5	1	4	24 h
Dixon 2019					5	4	3	3					23	18	20	11					16	16	15	12	N/A
El-Menyar 2020	5.2		9.7																						N/A
Howard 2017					1.5	1.9	1	1.6					12.5	11.5	8.4	8.4	3.2	5.7	2.2	4.8	11.1	9.9	7.7	8.2	24 h
Johnston 2018	34.5	25	5.2	14.5	2	1.9	0.3	1.5	1.3	3.1	0.3	2.0	13.1	8.6	2.2	5	6.5	9	0.8	4	11.6	7.6	1.7	4.3	24 h
Khan 2018					13		10						15		12						15		10		24 h
Lewis 2016													17		7										24 h
Moore 2017					3		2						28		19		2		0		15		8		N/A
Morrison 2012					3.2	2.4	3.6	3.6					21	12.8	22.5	15.9	1.6	2.6	0.7	1.6	18.4	11.5	19.6	14.3	24h
Morrison 2013					0.7	1.1	0.2	0.8					8	6.2	5.3	7.9					7.3	5.3	3.7	5.9	24 h
Morte 2019	18.3	14.2	16.5	16.3																					
Myers 2019					1.18	2.17	0.43	1.43					4.43	5.57	2.53	3.35					2.77	5.14	1.44	3.37	N/A
Neeki 2018	1		3																						D
Neeki 2020	4		7																						D

© 2022 Karl V et al. *JAMA Network Open*.

Shiraishi 2017													4.5		4						4.7		4		24 h
Swendsen 2013					1		0						7		2						3		0		N/A
Wafaisade 2016					0.1	0.7	0.1	0.9					1.6	4.3	2	5.8					1.2	3.8	1.4	4.6	ER to ICU

unit.e: Number of units in the experimental group; sd.e: Standard deviation in the experimental group; unit.c: Number of units in the control group; sd.c: Standard deviation in the control group; D: Discharge; ER to ICU: Time between emergeny room and intensive care unit; PLT: Platelets; WB: Whole blood; PRBC: Packed red blood cells; Cryo: Cryoprecipitate; FFP: Fresh frozen plasma

© 2022 Karl V et al. *JAMA Network Open*.

eFigure 2. **24-Hour Mortality**

Study	Experimental Events	Experimental Total	Control Events	Control Total	Risk Ratio IV, 95% CI
Guyette 2020 RCT	16	447	17	453	0.95 [0.49; 1.86]
Glover 2019	14	333	27	706	1.10 [0.58; 2.07]
Harvin 2015	33	98	93	934	3.38 [2.41; 4.74]
Howard 2017	20	373	38	657	0.93 [0.55; 1.57]
Khan 2018	8	31	24	62	0.67 [0.34; 1.31]
Luehr 2017	1	53	11	62	0.11 [0.01; 0.80]
Morrison 2012	12	125	29	196	0.65 [0.34; 1.22]
Neeki 2018	7	362	13	362	0.54 [0.22; 1.33]
Neeki 2020	3	280	3	280	1.00 [0.20; 4.91]
Swendsen 2013	3	52	13	74	0.33 [0.10; 1.09]
Wafaisade 2016	15	258	32	258	0.47 [0.26; 0.84]
**Prediction interval**					**[0.17; 3.63]**

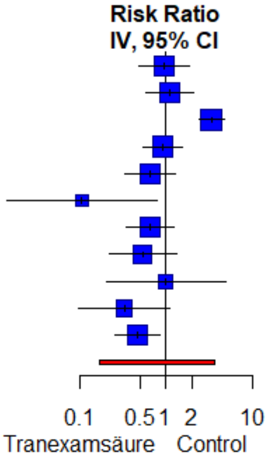

eFigure 3. **24-Hour Mortality in Observational Studies Excluding Studies at High Risk for Confounding Bias**

Study	Experimental Events	Experimental Total	Control Events	Control Total	Weight	Risk Ratio IV, Random, 95% CI
Howard 2017	20	373	38	657	35.0%	0.93 [0.55; 1.57]
Khan 2018	8	31	24	62	21.4%	0.67 [0.34; 1.31]
Neeki 2018	7	362	13	362	11.8%	0.54 [0.22; 1.33]
Neeki 2020	3	280	3	280	3.8%	1.00 [0.20; 4.91]
Wafaisade 2016	15	258	32	258	28.0%	0.47 [0.26; 0.84]
**Total (95% CI)**		**1304**		**1619**	**100.0%**	**0.67 [0.45; 1.01]**
**Prediction interval**						**[0.42; 1.07]**

Heterogeneity: $Tau^2 = 0$; $Chi^2 = 3.34$, df = 4 (P = 0.50); $I^2 = 0\%$

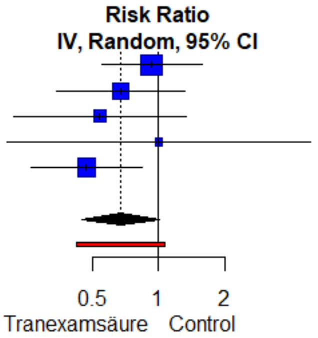

eFigure 4. **1-Month Mortality in RCTs**

Study	Experimental Events	Experimental Total	Control Events	Control Total	Weight	Risk Ratio IV, Random, 95% CI
Chakroun□Walha 2019 RCT	27	96	19	84	1.0%	1.24 [0.75; 2.07]
CRASH-2 RCT 2010	1463	10060	1613	10067	59.5%	0.91 [0.85; 0.97]
CRASH-3 2019 RCT	855	4613	892	4514	35.5%	0.94 [0.86; 1.02]
Guyette 2020 RCT	36	442	45	452	1.4%	0.82 [0.54; 1.24]
Rowell 2020 RCT	93	603	50	285	2.6%	0.88 [0.64; 1.20]
**Total (95% CI)**		**15814**		**15402**	**100.0%**	**0.92 [0.87; 0.97]**
**Prediction interval**						**[0.87; 0.97]**

Heterogeneity: $Tau^2 = 0$; $Chi^2 = 2.10$, df = 4 (P = 0.72); $I^2 = 0\%$

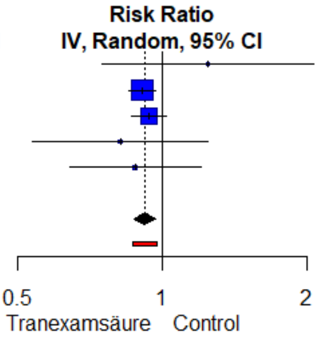

© 2022 Karl V et al. *JAMA Network Open*.

eFigure 5. **1-Month Mortality in Observational Studies Excluding Studies at High Risk for Confounding Bias**

Study	Experimental Events	Experimental Total	Control Events	Control Total	Weight	Risk Ratio IV, Random, 95% CI
Boutonnet 2018	94	403	65	281	17.8%	1.01 [0.76; 1.33]
Dixon 2019	24	62	31	62	14.6%	0.77 [0.52; 1.16]
Howard 2017	27	373	59	657	13.7%	0.81 [0.52; 1.25]
Khan 2018	14	31	31	62	13.1%	0.90 [0.57; 1.43]
Neeki 2018	13	362	30	362	9.6%	0.43 [0.23; 0.82]
Neeki 2020	3	280	14	280	3.7%	0.21 [0.06; 0.74]
Shiraishi 2017	25	250	46	250	13.3%	0.54 [0.35; 0.86]
Wafaisade 2016	36	258	42	258	14.3%	0.86 [0.57; 1.29]
**Total (95% CI)**		**2019**		**2212**	**100.0%**	**0.73 [0.53; 1.00]**
**Prediction interval**						**[0.34; 1.55]**

Heterogeneity: $Tau^2 = 0.0789$; $Chi^2 = 13.80$, df = 7 (P = 0.05); $I^2 = 49\%$

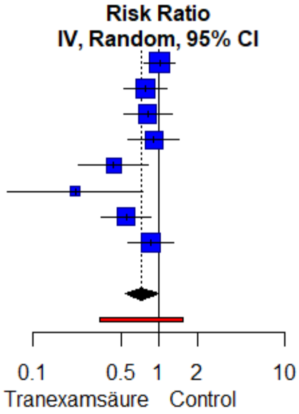

eFigure 6. **1-Month Mortality in Multiple Trauma**

Study	Experimental Events	Experimental Total	Control Events	Control Total	Weight	Risk Ratio IV, Random, 95% CI
CRASH-2 2010 RCT	1463	10060	1613	10067	21.0%	0.91 [0.85; 0.97]
Guyette 2020 RCT	17	352	25	374	6.2%	0.72 [0.40; 1.31]
Boutonnet 2018	94	403	65	281	14.1%	1.01 [0.76; 1.33]
Dixon 2019	24	62	31	62	10.3%	0.77 [0.52; 1.16]
Howard 2017	27	373	59	657	9.3%	0.81 [0.52; 1.25]
Khan 2018	14	31	31	62	8.8%	0.90 [0.57; 1.43]
Luehr 2017	7	53	13	62	3.7%	0.63 [0.27; 1.46]
Neeki 2018	13	362	30	362	5.7%	0.43 [0.23; 0.82]
Neeki 2020	3	280	14	280	1.9%	0.21 [0.06; 0.74]
Shiraishi 2017	25	250	46	250	8.9%	0.54 [0.35; 0.86]
Wafaisade 2016	36	258	42	258	10.0%	0.86 [0.57; 1.29]
**Total (95% CI)**		**12484**		**12715**	**100.0%**	**0.77 [0.63; 0.94]**
**Prediction interval**						**[0.48; 1.25]**

Heterogeneity: $Tau^2 = 0.0379$; $Chi^2 = 17.48$, df = 10 (P = 0.06); $I^2 = 43\%$

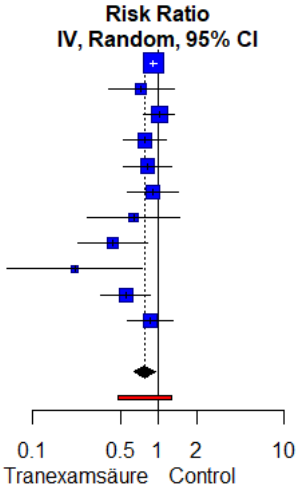

eFigure 7. **1-Month Mortality in TBI**

Study	Experimental Events	Experimental Total	Control Events	Control Total	Weight	Risk Ratio IV, Random, 95% CI
Chakroun□Walha 2019 RCT	27	96	19	84	2.4%	1.24 [0.75; 2.07]
CRASH-3 2019 RCT	855	4613	892	4514	89.0%	0.94 [0.86; 1.02]
Guyette 2020 RCT	19	90	20	78	2.1%	0.82 [0.48; 1.43]
Rowell 2020 RCT	93	603	50	285	6.4%	0.88 [0.64; 1.20]
**Total (95% CI)**		**5402**		**4961**	**100.0%**	**0.94 [0.85; 1.03]**
**Prediction interval**						**[0.83; 1.06]**

Heterogeneity: $Tau^2 = 0$; $Chi^2 = 1.56$, df = 3 (P = 0.67); $I^2 = 0\%$

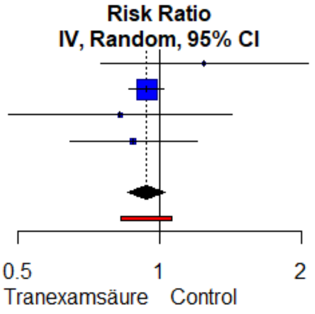

© 2022 Karl V et al. *JAMA Network Open*.

eFigure 8. **1-Month Mortality in Standard Protocol Tranexamic Acid Administration**

Study	Experimental Events	Experimental Total	Control Events	Control Total	Weight	Risk Ratio IV, Random, 95% CI
Chakroun-Walha 2019 RCT	27	96	19	84	11.7%	1.24 [0.75; 2.07]
CRASH-2 2010 RCT	1463	10060	1613	10067	18.3%	0.91 [0.85; 0.97]
CRASH-3 2019 RCT	855	4613	892	4514	18.2%	0.94 [0.86; 1.02]
Guyette 2020 RCT	11	141	45	452	9.8%	0.78 [0.42; 1.47]
Rowell 2020 RCT	53	285	50	285	14.5%	1.06 [0.75; 1.50]
Dixon 2019	24	62	31	62	13.6%	0.77 [0.52; 1.16]
Neeki 2018	13	362	30	362	9.7%	0.43 [0.23; 0.82]
Neeki 2020	3	280	14	280	4.2%	0.21 [0.06; 0.74]
**Total (95% CI)**		**15899**		**16106**	**100.0%**	**0.82 [0.58; 1.16]**
**Prediction interval**						**[0.33; 2.03]**

Heterogeneity: $Tau^2 = 0.1165$; $Chi^2 = 14.00$, df = 7 (P = 0.05); $I^2 = 50\%$

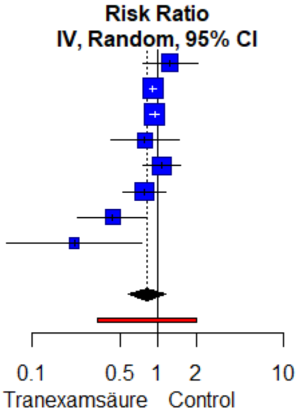

eFigure 9. **1-Month Mortality in Nonstandard Protocol Tranexamic Acid Administration**

Study	Experimental Events	Experimental Total	Control Events	Control Total	Weight	Risk Ratio IV, Random, 95% CI
Guyette 2020 RCT	25	301	45	452	9.9%	0.83 [0.52; 1.33]
Rowell 2020 RCT	40	318	50	285	14.6%	0.72 [0.49; 1.05]
Boutonnet 2018	94	403	65	281	28.0%	1.01 [0.76; 1.33]
Howard 2017	27	373	59	657	11.2%	0.81 [0.52; 1.25]
Khan 2018	14	31	31	62	10.1%	0.90 [0.57; 1.43]
Luehr 2017	7	53	13	62	3.0%	0.63 [0.27; 1.46]
Shiraishi 2017	25	250	46	250	10.4%	0.54 [0.35; 0.86]
Wafaisade 2016	36	258	42	258	12.8%	0.86 [0.57; 1.29]
**Total (95% CI)**		**1987**		**2307**	**100.0%**	**0.82 [0.69; 0.97]**
**Prediction interval**						**[0.69; 0.98]**

Heterogeneity: $Tau^2 = 0$; $Chi^2 = 6.36$, df = 7 (P = 0.50); $I^2 = 0\%$

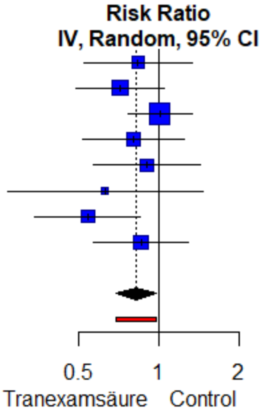

© 2022 Karl V et al. *JAMA Network Open*.

eFigure 10. **Overall Mortality**

Study	Experimental Events	Experimental Total	Control Events	Control Total	Risk Ratio IV, 95% CI
Chakroun-Walha 2019 RCT	27	96	19	84	1.24 [0.75; 2.07]
CRASH-2 2010 RCT	1463	10060	1613	10067	0.91 [0.85; 0.97]
CRASH-3 2019 RCT	855	4613	892	4514	0.94 [0.86; 1.02]
Guyette 2020 RCT	36	442	45	452	0.82 [0.54; 1.24]
Rowell 2020 RCT	101	551	54	272	0.92 [0.69; 1.24]
Yutthakasemsunt 2013 RCT	12	120	17	118	0.69 [0.35; 1.39]
Bardes 2017	4	32	23	119	0.65 [0.24; 1.74]
Boutonnet 2018	94	403	65	281	1.01 [0.76; 1.33]
Cole 2015	17	160	18	225	1.33 [0.71; 2.50]
Dixon 2019	24	62	31	62	0.77 [0.52; 1.16]
El-Menyar 2020	25	102	30	102	0.83 [0.53; 1.31]
Glover 2019	47	333	106	706	0.94 [0.68; 1.29]
Harvin 2015	39	98	159	934	2.34 [1.76; 3.10]
Howard 2017	27	373	59	657	0.81 [0.52; 1.25]
Johnston 2018	1	146	3	309	0.71 [0.07; 6.72]
Khan 2018	14	31	31	62	0.90 [0.57; 1.43]
Lewis 2016	10	335	11	626	1.70 [0.73; 3.96]
Luehr 2017	7	53	13	62	0.63 [0.27; 1.46]
Moore 2017	13	26	35	206	2.94 [1.81; 4.80]
Morrison 2012	18	125	55	196	0.51 [0.32; 0.83]
Morrison 2013	27	148	179	758	0.77 [0.54; 1.11]
Morte 2019	0	46	5	46	0.09 [0.01; 1.60]
Myers 2019	53	189	28	189	1.89 [1.25; 2.86]
Neeki 2018	13	362	30	362	0.43 [0.23; 0.82]
Neeki 2020	3	280	14	280	0.21 [0.06; 0.74]
Shiraishi 2017	25	250	46	250	0.54 [0.35; 0.86]
Swendsen 2013	9	52	17	74	0.75 [0.36; 1.56]
Valle 2014	47	150	35	150	1.34 [0.92; 1.95]
Wafaisade 2016	38	258	42	258	0.90 [0.60; 1.35]
Walker 2020	1	14	4	57	1.02 [0.12; 8.41]
**Prediction interval**					**[0.40; 2.17]**

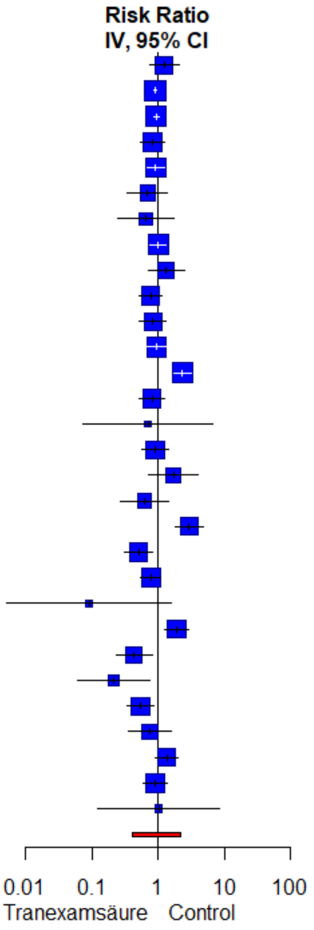

eFigure 11. **Overall Mortality in RCTs**

Study	Experimental Events	Experimental Total	Control Events	Control Total	Weight	Risk Ratio IV, Random, 95% CI
Chakroun_Walha 2019 (RCT)	27	96	19	84	1.0%	1.24 [0.75; 2.07]
CRASH-2 (RCT)	1463	10060	1613	10067	59.0%	0.91 [0.85; 0.97]
CRASH-3 (RCT)	855	4613	892	4514	35.2%	0.94 [0.86; 1.02]
Guyette 2020 (RCT)	36	442	45	452	1.4%	0.82 [0.54; 1.24]
Rowell 2020 (RCT)	101	551	54	272	2.8%	0.92 [0.69; 1.24]
Yutthakasemsunt 2013 (RCT)	12	120	17	118	0.5%	0.69 [0.35; 1.39]
**Total (95% CI)**		**15882**		**15507**	**100.0%**	**0.92 [0.86; 0.98]**
**Prediction interval**						**[0.86; 0.99]**

Heterogeneity: $Tau^2 = 0$; $Chi^2 = 2.65$, df = 5 (P = 0.75); $I^2 = 0\%$

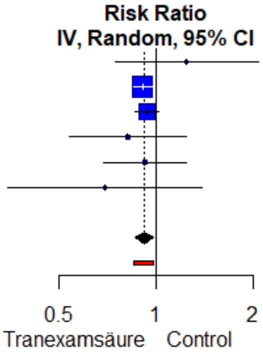

© 2022 Karl V et al. *JAMA Network Open*.

eFigure 12. **Overall Mortality in Observational Studies Excluding Studies at High Risk for Confounding Bias**

Study	Experimental Events	Experimental Total	Control Events	Control Total	Risk Ratio IV, 95% CI
Bardes 2017	4	32	23	119	0.65 [0.24; 1.74]
Boutonnet 2018	94	403	65	281	1.01 [0.76; 1.33]
Cole 2015	17	160	18	225	1.33 [0.71; 2.50]
Dixon 2019	24	62	31	62	0.77 [0.52; 1.16]
El-Menyar 2020	25	102	30	102	0.83 [0.53; 1.31]
Howard 2017	27	373	59	657	0.81 [0.52; 1.25]
Khan 2018	14	31	31	62	0.90 [0.57; 1.43]
Morrison 2013	27	148	179	758	0.77 [0.54; 1.11]
Morte 2019	0	46	5	46	0.09 [0.01; 1.60]
Myers 2019	53	189	28	189	1.89 [1.25; 2.86]
Neeki 2018	13	362	30	362	0.43 [0.23; 0.82]
Neeki 2020	3	280	14	280	0.21 [0.06; 0.74]
Shiraishi 2017	25	250	46	250	0.54 [0.35; 0.86]
Valle 2014	47	150	35	150	1.34 [0.92; 1.95]
Wafaisade 2016	38	258	42	258	0.90 [0.60; 1.35]
**Prediction interval**					**[0.35; 2.05]**

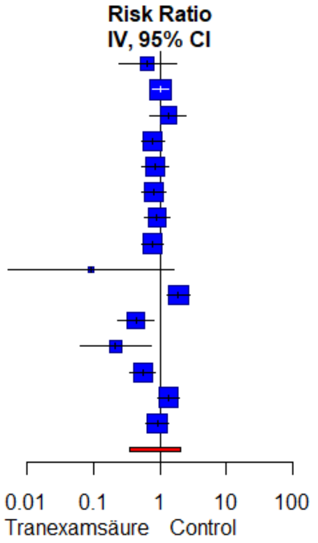

eFigure 13. **Overall Mortality in Multiple Trauma**

Study	Experimental Events	Experimental Total	Control Events	Control Total	Risk Ratio IV, 95% CI
CRASH-2 2010 RCT	1463	10060	1613	10067	0.91 [0.85; 0.97]
Guyette 2020 RCT	17	352	25	374	0.72 [0.40; 1.31]
Bardes 2017	4	32	23	119	0.65 [0.24; 1.74]
Boutonnet 2018	94	403	65	281	1.01 [0.76; 1.33]
Cole 2015	17	160	18	225	1.33 [0.71; 2.50]
Dixon 2019	24	62	31	62	0.77 [0.52; 1.16]
El-Menyar 2020	25	102	30	102	0.83 [0.53; 1.31]
Glover 2019	47	333	106	706	0.94 [0.68; 1.29]
Harvin 2015	39	98	159	934	2.34 [1.76; 3.10]
Howard 2017	20	373	38	657	0.93 [0.55; 1.57]
Johnston 2018	1	146	3	309	0.71 [0.07; 6.72]
Khan 2018	14	31	31	62	0.90 [0.57; 1.43]
Lewis 2016	10	335	11	626	1.70 [0.73; 3.96]
Luehr 2017	7	53	13	62	0.63 [0.27; 1.46]
Moore 2017	13	26	35	206	2.94 [1.81; 4.80]
Morrison 2012	18	125	55	196	0.51 [0.32; 0.83]
Morrison 2013	27	148	179	758	0.77 [0.54; 1.11]
Myers 2019	53	189	28	189	1.89 [1.25; 2.86]
Neeki 2018	13	362	30	362	0.43 [0.23; 0.82]
Neeki 2020	3	280	14	280	0.21 [0.06; 0.74]
Shiraishi 2017	25	250	46	250	0.54 [0.35; 0.86]
Swendsen 2013	9	52	17	74	0.75 [0.36; 1.56]
Valle 2014	25	109	14	105	1.72 [0.95; 3.12]
Wafaisade 2016	38	258	42	258	0.90 [0.60; 1.35]
**Prediction interval**					**[0.36; 2.47]**

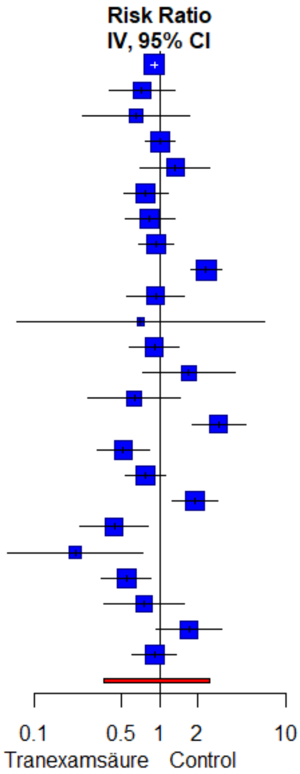

© 2022 Karl V et al. *JAMA Network Open*.

eFigure 14. **Overall Mortality in TBI**

Study	Experimental Events	Experimental Total	Control Events	Control Total	Weight	Risk Ratio IV, Random, 95% CI
Chakroun□Walha 2019 RCT	27	96	19	84	2.4%	1.24 [0.75; 2.07]
CRASH-3 2019 RCT	855	4613	892	4514	85.9%	0.94 [0.86; 1.02]
Guyette 2020 RCT	19	90	20	78	2.0%	0.82 [0.48; 1.43]
Rowell 2020 RCT	101	551	54	272	6.9%	0.92 [0.69; 1.24]
Yutthakasemsunt 2013 RCT	12	120	17	118	1.3%	0.69 [0.35; 1.39]
Morte 2019	0	46	5	46	0.1%	0.09 [0.01; 1.60]
Valle 2014	13	32	9	34	1.2%	1.53 [0.76; 3.09]
Walker 2020	1	14	4	57	0.1%	1.02 [0.12; 8.41]
**Total (95% CI)**		**5562**		**5203**	**100.0%**	**0.94 [0.86; 1.03]**
**Prediction interval**						**[0.86; 1.03]**

Heterogeneity: Tau^2 = 0; Chi^2 = 6.58, df = 7 (P = 0.47); I^2 = 0%

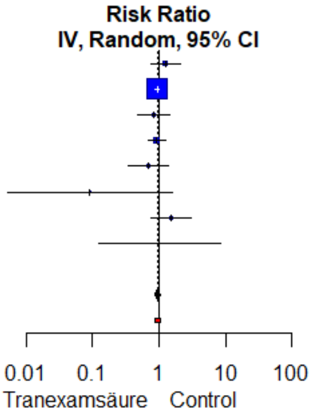

eFigure 15. **Overall Mortality in In-hospital Tranexamic Acid Administration**

Study	Experimental Events	Experimental Total	Control Events	Control Total	Weight	Risk Ratio IV, Random, 95% CI
Chakroun□Walha 2019 RCT	27	96	19	84	20.2%	1.24 [0.75; 2.07]
CRASH-2 2010 RCT	1463	10060	1613	10067	27.3%	0.91 [0.85; 0.97]
CRASH-3 2019 RCT	855	4613	892	4514	27.2%	0.94 [0.86; 1.02]
Yutthakasemsunt 2013 RCT	12	120	17	118	16.5%	0.69 [0.35; 1.39]
Neeki 2020	3	280	14	280	8.8%	0.21 [0.06; 0.74]
**Total (95% CI)**		**15169**		**15063**	**100.0%**	**0.82 [0.44; 1.54]**
**Prediction interval**						**[0.17; 3.89]**

Heterogeneity: Tau^2 = 0.1871; Chi^2 = 7.68, df = 4 (P = 0.10); I^2 = 48%

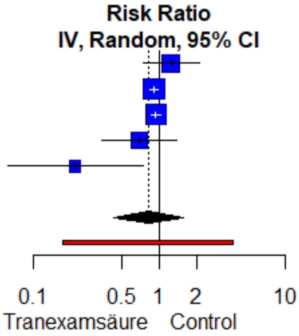

eFigure 16. **Overall Mortality in Prehospital Tranexamic Acid Administration**

Study	Experimental Events	Experimental Total	Control Events	Control Total	Weight	Risk Ratio IV, Random, 95% CI
Guyette 2020 RCT	36	442	45	452	19.8%	0.82 [0.54; 1.24]
Rowell 2020 RCT	101	551	54	272	32.0%	0.92 [0.69; 1.24]
El-Menyar 2020	25	102	30	102	17.4%	0.83 [0.53; 1.31]
Neeki 2018	13	362	30	362	9.9%	0.43 [0.23; 0.82]
Wafaisade 2016	38	258	42	258	20.9%	0.90 [0.60; 1.35]
**Total (95% CI)**		**1715**		**1446**	**100.0%**	**0.82 [0.61; 1.10]**
**Prediction interval**						**[0.49; 1.36]**

Heterogeneity: Tau^2 = 0.0136; Chi^2 = 4.71, df = 4 (P = 0.32); I^2 = 15%

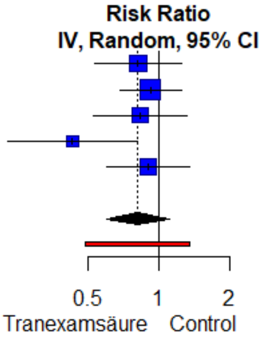

© 2022 Karl V et al. *JAMA Network Open*.

eFigure 17. **Overall Mortality in Standard Protocol Tranexamic Acid Administration**

Study	Experimental Events	Experimental Total	Control Events	Control Total	Risk Ratio IV, 95% CI
Chakroun-Walha 2019 RCT	27	96	19	84	1.24 [0.75; 2.07]
CRASH-2 2010 RCT	1463	10060	1613	10067	0.91 [0.85; 0.97]
CRASH-3 2019 RCT	855	4613	892	4514	0.94 [0.86; 1.02]
Guyette 2020 RCT	11	141	45	452	0.78 [0.42; 1.47]
Rowell 2020 RCT	55	262	54	272	1.06 [0.76; 1.48]
Yutthakasemsunt 2013 RCT	12	120	17	118	0.69 [0.35; 1.39]
Bardes 2017	4	32	23	119	0.65 [0.24; 1.74]
Cole 2015	17	160	18	225	1.33 [0.71; 2.50]
Dixon 2019	24	62	31	62	0.77 [0.52; 1.16]
Morte 2019	0	46	5	46	0.09 [0.01; 1.60]
Neeki 2018	13	362	30	362	0.43 [0.23; 0.82]
Neeki 2020	3	280	14	280	0.21 [0.06; 0.74]
Swendsen 2013	9	52	17	74	0.75 [0.36; 1.56]
Valle 2014	47	150	35	150	1.34 [0.92; 1.95]
**Prediction interval**					**[0.42; 1.75]**

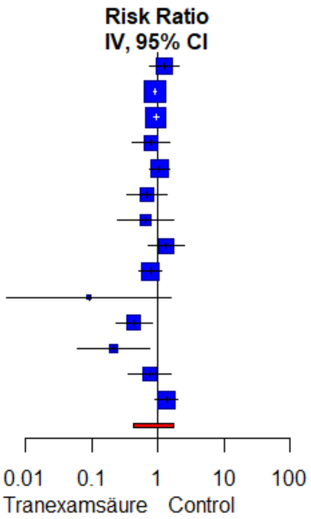

eFigure 18. **Overall Mortality in Nonstandard Tranexamic Acid Administration**

Study	Experimental Events	Experimental Total	Control Events	Control Total	Risk Ratio IV, 95% CI
Guyette 2020 RCT	25	301	45	452	0.83 [0.52; 1.33]
Rowell 2020 RCT	46	289	54	272	0.80 [0.56; 1.15]
Boutonnet 2018	94	403	65	281	1.01 [0.76; 1.33]
El-Menyar 2020	25	102	30	102	0.83 [0.53; 1.31]
Glover 2019	47	333	106	706	0.94 [0.68; 1.29]
Harvin 2015	39	98	159	934	2.34 [1.76; 3.10]
Howard 2017	27	373	59	657	0.81 [0.52; 1.25]
Johnston 2018	1	146	3	309	0.71 [0.07; 6.72]
Khan 2018	14	31	31	62	0.90 [0.57; 1.43]
Lewis 2016	10	335	11	626	1.70 [0.73; 3.96]
Luehr 2017	7	53	13	62	0.63 [0.27; 1.46]
Moore 2017	13	26	35	206	2.94 [1.81; 4.80]
Morrison 2012	18	125	55	196	0.51 [0.32; 0.83]
Morrison 2013	27	148	179	758	0.77 [0.54; 1.11]
Myers 2019	53	189	28	189	1.89 [1.25; 2.86]
Shiraishi 2017	25	250	46	250	0.54 [0.35; 0.86]
Wafaisade 2016	38	258	42	258	0.90 [0.60; 1.35]
Walker 2020	1	14	4	57	1.02 [0.12; 8.41]
**Prediction interval**					**[0.41; 2.49]**

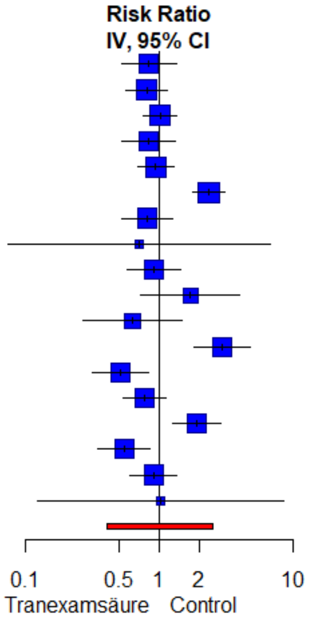

© 2022 Karl V et al. *JAMA Network Open*.

eFigure 19. **Thromboembolic Events**

Study	Experimental Events	Experimental Total	Control Events	Control Total	Risk Ratio IV, 95% CI
Chakroun-Walha 2019 RCT	14	96	5	84	2.45 [0.92; 6.52]
CRASH-2 2010 RCT	168	10060	201	10067	0.84 [0.68; 1.02]
CRASH-3 2019 RCT	101	6359	102	6280	0.98 [0.74; 1.28]
Guyette 2020 RCT	25	447	14	453	1.81 [0.95; 3.44]
Rowell 2020 RCT	44	657	30	309	0.69 [0.44; 1.08]
Yutthakasemsunt 2013 RCT	0	120	3	118	0.14 [0.01; 2.69]
Chan 2019	2	81	35	570	0.40 [0.10; 1.64]
Cole 2015	16	160	15	225	1.50 [0.76; 2.94]
Dixon 2019	6	62	8	62	0.75 [0.28; 2.04]
El-Menyar 2020	4	102	2	102	2.00 [0.37; 10.68]
Glover 2019	21	333	27	706	1.65 [0.95; 2.87]
Harvin 2015	6	98	41	934	1.39 [0.61; 3.20]
Howard 2017	11	373	7	657	2.77 [1.08; 7.08]
Johnston 2018	50	146	21	309	5.04 [3.15; 8.06]
Khan 2018	4	31	8	62	1.00 [0.33; 3.07]
Luehr 2017	7	53	11	62	0.74 [0.31; 1.78]
Morrison 2012	6	125	1	196	9.41 [1.15; 77.22]
Morte 2019	2	46	1	46	2.00 [0.19; 21.30]
Myers 2019	29	189	14	189	2.07 [1.13; 3.79]
Neeki 2018	2	362	2	362	1.00 [0.14; 7.06]
Neeki 2020	2	280	2	280	1.00 [0.14; 7.05]
Shiraishi 2017	3	250	5	250	0.60 [0.14; 2.48]
Swendsen 2013	8	52	0	74	24.12 [1.42; 408.88]
Wafaisade 2016	4	71	10	121	0.68 [0.22; 2.09]
**Prediction interval**					**[0.42; 4.35]**

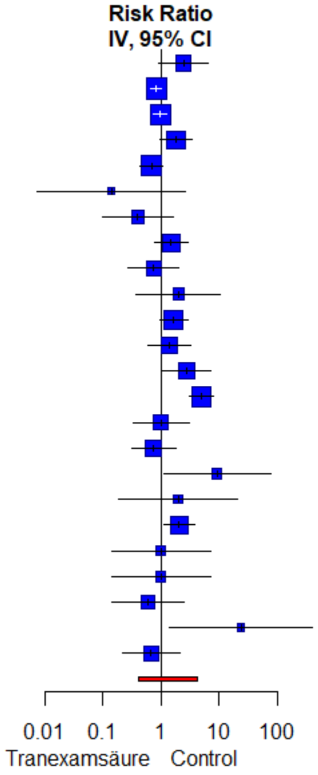

eFigure 20. **Thromboembolic Events in RCTs**

Study	Experimental Events	Experimental Total	Control Events	Control Total	Risk Ratio IV, 95% CI
Chakroun_Walha 2019 (RCT)	14	96	5	84	2.45 [0.92; 6.52]
CRASH-2 (RCT)	168	10060	201	10067	0.84 [0.68; 1.02]
CRASH-3 (RCT)	101	6359	102	6280	0.98 [0.74; 1.28]
Guyette 2020 (RCT)	25	447	14	453	1.81 [0.95; 3.44]
Rowell 2020 (RCT)	44	657	30	309	0.69 [0.44; 1.08]
Yutthakasemsunt 2013 (RCT)	0	120	3	118	0.14 [0.01; 2.69]
**Prediction interval**					**[0.24; 4.41]**

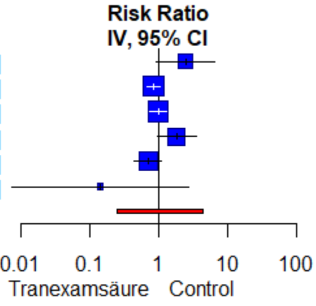

© 2022 Karl V et al. *JAMA Network Open*.

eFigure 21. **Thromboembolic Events in Observational Studies Excluding Studies at High Risk for Confounding Bias**

Study	Experimental Events	Experimental Total	Control Events	Control Total	Weight	Risk Ratio IV, Random, 95% CI
Chan 2019	2	81	35	570	5.1%	0.40 [0.10; 1.64]
Cole 2015	16	160	15	225	19.6%	1.50 [0.76; 2.94]
Dixon 2019	6	62	8	62	9.7%	0.75 [0.28; 2.04]
El-Menyar 2020	4	102	2	102	3.6%	2.00 [0.37; 10.68]
Howard 2017	11	373	7	657	10.8%	2.77 [1.08; 7.08]
Khan 2018	4	31	8	62	7.8%	1.00 [0.33; 3.07]
Morte 2019	2	46	1	46	1.8%	2.00 [0.19; 21.30]
Myers 2019	29	189	14	189	23.5%	2.07 [1.13; 3.79]
Neeki 2018	2	362	2	362	2.7%	1.00 [0.14; 7.06]
Neeki 2020	2	280	2	280	2.7%	1.00 [0.14; 7.05]
Shiraishi 2017	3	250	5	250	5.0%	0.60 [0.14; 2.48]
Wafaisade 2016	4	71	10	121	7.8%	0.68 [0.22; 2.09]
**Total (95% CI)**		**2007**		**2926**	**100.0%**	**1.31 [0.91; 1.88]**
**Prediction interval**						**[0.81; 2.12]**

Heterogeneity: $Tau^2 = 0.0195$; $Chi^2 = 11.89$, df = 11 (P = 0.37); $I^2 = 7\%$

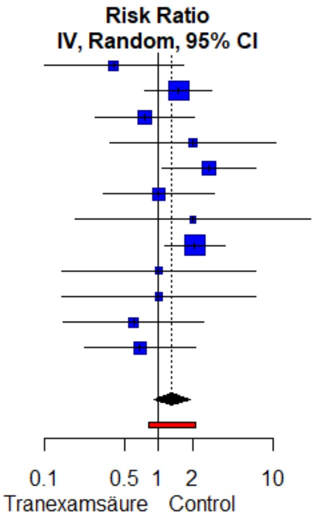

eFigure 22. **Thromboembolic Events in Multiple Trauma**

Study	Experimental Events	Experimental Total	Control Events	Control Total	Risk Ratio IV, 95% CI
CRASH-2 2010 RCT	168	10060	201	10067	0.84 [0.68; 1.02]
Guyette 2020 RCT	25	447	14	453	1.81 [0.95; 3.44]
Cole 2015	16	160	15	225	1.50 [0.76; 2.94]
Dixon 2019	6	62	8	62	0.75 [0.28; 2.04]
El-Menyar 2020	4	102	2	102	2.00 [0.37; 10.68]
Glover 2019	21	333	27	706	1.65 [0.95; 2.87]
Harvin 2015	6	98	41	934	1.39 [0.61; 3.20]
Howard 2017	11	373	7	657	2.77 [1.08; 7.08]
Johnston 2018	50	146	21	309	5.04 [3.15; 8.06]
Khan 2018	4	31	8	62	1.00 [0.33; 3.07]
Luehr 2017	7	53	11	62	0.74 [0.31; 1.78]
Morrison 2012	6	125	1	196	9.41 [1.15; 77.22]
Myers 2019	29	189	14	189	2.07 [1.13; 3.79]
Neeki 2018	2	362	2	362	1.00 [0.14; 7.06]
Neeki 2020	2	280	2	280	1.00 [0.14; 7.05]
Shiraishi 2017	3	250	5	250	0.60 [0.14; 2.48]
Swendsen 2013	8	52	0	74	24.12 [1.42; 408.88]
Wafaisade 2016	4	71	10	121	0.68 [0.22; 2.09]
**Prediction interval**					**[0.47; 4.91]**

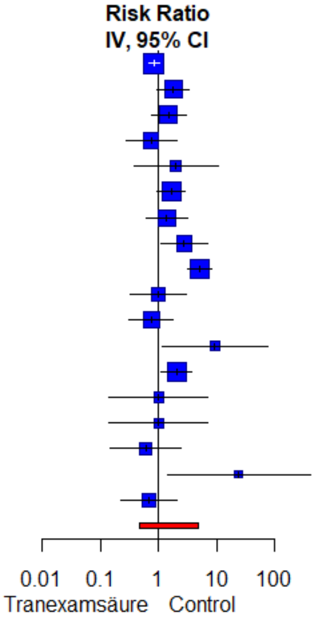

© 2022 Karl V et al. *JAMA Network Open*.

eFigure 23. **Thromboembolic Events in TBI**

Study	Experimental Events	Experimental Total	Control Events	Control Total	Weight	Risk Ratio IV, Random, 95% CI
Chakroun□Walha 2019 RCT	14	96	5	84	17.4%	2.45 [0.92; 6.52]
CRASH-3 2019 RCT	101	6359	102	6280	33.4%	0.98 [0.74; 1.28]
Rowell 2020 RCT	44	657	30	309	29.6%	0.69 [0.44; 1.08]
Yutthakasemsunt 2013 RCT	0	120	3	118	3.3%	0.14 [0.01; 2.69]
Chan 2019	2	81	35	570	11.2%	0.40 [0.10; 1.64]
Morte 2019	2	46	1	46	4.9%	2.00 [0.19; 21.30]
**Total (95% CI)**		**7359**		**7407**	**100.0%**	**0.91 [0.43; 1.91]**
**Prediction interval**						**[0.19; 4.31]**

Heterogeneity: $Tau^2 = 0.2302$; $Chi^2 = 8.96$, df = 5 (P = 0.11); $I^2 = 44\%$

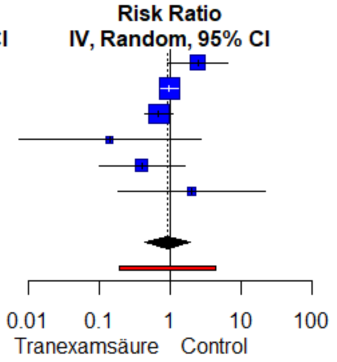

eFigure 24. **Thromboembolic Events in In-hospital Tranexamic Acid Administration**

Study	Experimental Events	Experimental Total	Control Events	Control Total	Weight	Risk Ratio IV, Random, 95% CI
Chakroun□Walha 2019 RCT	14	96	5	84	14.3%	2.45 [0.92; 6.52]
CRASH-2 2010 RCT	168	10060	201	10067	35.9%	0.84 [0.68; 1.02]
CRASH-3 2019 RCT	101	6359	102	6280	34.0%	0.98 [0.74; 1.28]
Yutthakasemsunt 2013 RCT	0	120	3	118	2.3%	0.14 [0.01; 2.69]
Chan 2019	2	81	35	570	8.6%	0.40 [0.10; 1.64]
Neeki 2020	2	280	2	280	5.0%	1.00 [0.14; 7.05]
**Total (95% CI)**		**16996**		**17399**	**100.0%**	**0.93 [0.51; 1.72]**
**Prediction interval**						**[0.27; 3.27]**

Heterogeneity: $Tau^2 = 0.1471$; $Chi^2 = 7.67$, df = 5 (P = 0.18); $I^2 = 35\%$

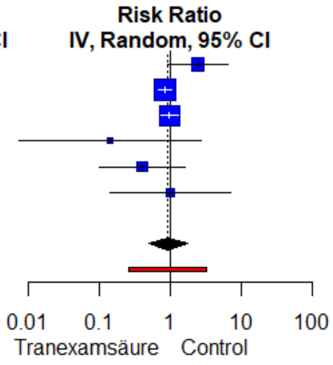

eFigure 25. **Thromboembolic Events in Prehospital Tranexamic Acid Administration**

Study	Experimental Events	Experimental Total	Control Events	Control Total	Weight	Risk Ratio IV, Random, 95% CI
Guyette 2020 RCT	25	447	14	453	30.4%	1.81 [0.95; 3.44]
Rowell 2020 RCT	44	657	30	309	43.9%	0.69 [0.44; 1.08]
El-Menyar 2020	4	102	2	102	6.8%	2.00 [0.37; 10.68]
Neeki 2018	2	362	2	362	5.2%	1.00 [0.14; 7.06]
Wafaisade 2016	4	71	10	121	13.7%	0.68 [0.22; 2.09]
**Total (95% CI)**		**1639**		**1347**	**100.0%**	**1.01 [0.53; 1.94]**
**Prediction interval**						**[0.32; 3.18]**

Heterogeneity: $Tau^2 = 0.0742$; $Chi^2 = 6.98$, df = 4 (P = 0.14); $I^2 = 43\%$

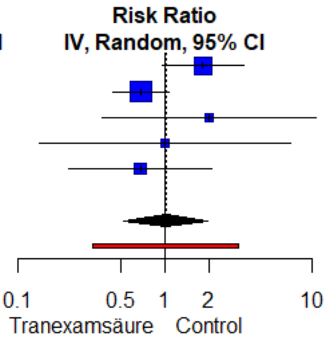

© 2022 Karl V et al. *JAMA Network Open*.

eFigure 26. **Thromboembolic Events in Standard Protocol Tranexamic Acid Administration**

Study	Experimental Events	Experimental Total	Control Events	Control Total	Risk Ratio IV, 95% CI
Chakroun-Walha 2019 RCT	14	96	5	84	2.45 [0.92; 6.52]
CRASH-2 2010 RCT	168	10060	201	10067	0.84 [0.68; 1.02]
CRASH-3 2019 RCT	101	6359	102	6280	0.98 [0.74; 1.28]
Rowell 2020 RCT	13	312	30	309	0.43 [0.23; 0.81]
Yutthakasemsunt 2013 RCT	0	120	3	118	0.14 [0.01; 2.69]
Cole 2015	16	160	15	225	1.50 [0.76; 2.94]
Dixon 2019	6	62	8	62	0.75 [0.28; 2.04]
Morte 2019	29	189	14	189	2.07 [1.13; 3.79]
Neeki 2018	2	362	2	362	1.00 [0.14; 7.06]
Neeki 2020	2	280	2	280	1.00 [0.14; 7.05]
Swendsen 2013	8	52	0	74	24.12 [1.42; 408.88]
**Prediction interval**					**[0.25; 4.78]**

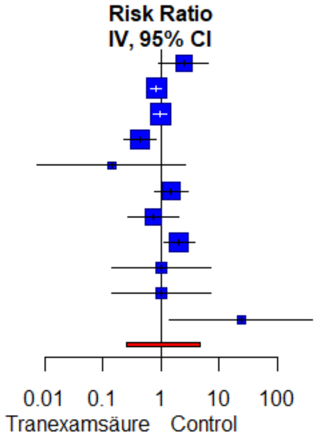

eFigure 27. **Thromboembolic Events in Nonstandard Protocol Tranexamic Acid Administration**

Study	Experimental Events	Experimental Total	Control Events	Control Total	Risk Ratio IV, 95% CI
Guyette 2020 RCT	25	447	14	453	1.81 [0.95; 3.44]
Rowell 2020 RCT	31	345	30	309	0.93 [0.57; 1.49]
Chan 2019	2	81	35	570	0.40 [0.10; 1.64]
El-Menyar 2020	4	102	2	102	2.00 [0.37; 10.68]
Glover 2019	21	333	27	706	1.65 [0.95; 2.87]
Harvin 2015	6	98	41	934	1.39 [0.61; 3.20]
Howard 2017	11	373	7	657	2.77 [1.08; 7.08]
Johnston 2018	50	146	21	309	5.04 [3.15; 8.06]
Khan 2018	4	31	8	62	1.00 [0.33; 3.07]
Luehr 2017	7	53	11	62	0.74 [0.31; 1.78]
Morrison 2012	6	125	1	196	9.41 [1.15; 77.22]
Myers 2019	29	189	14	189	2.07 [1.13; 3.79]
Shiraishi 2017	3	250	5	250	0.60 [0.14; 2.48]
Wafaisade 2016	9	27	25	94	1.25 [0.67; 2.35]
**Prediction interval**					**[0.45; 5.35]**

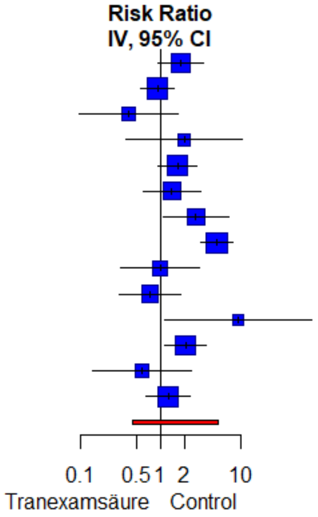

© 2022 Karl V et al. *JAMA Network Open*.

eFigure 28. **Funnel Plot for 1-Month Mortality**

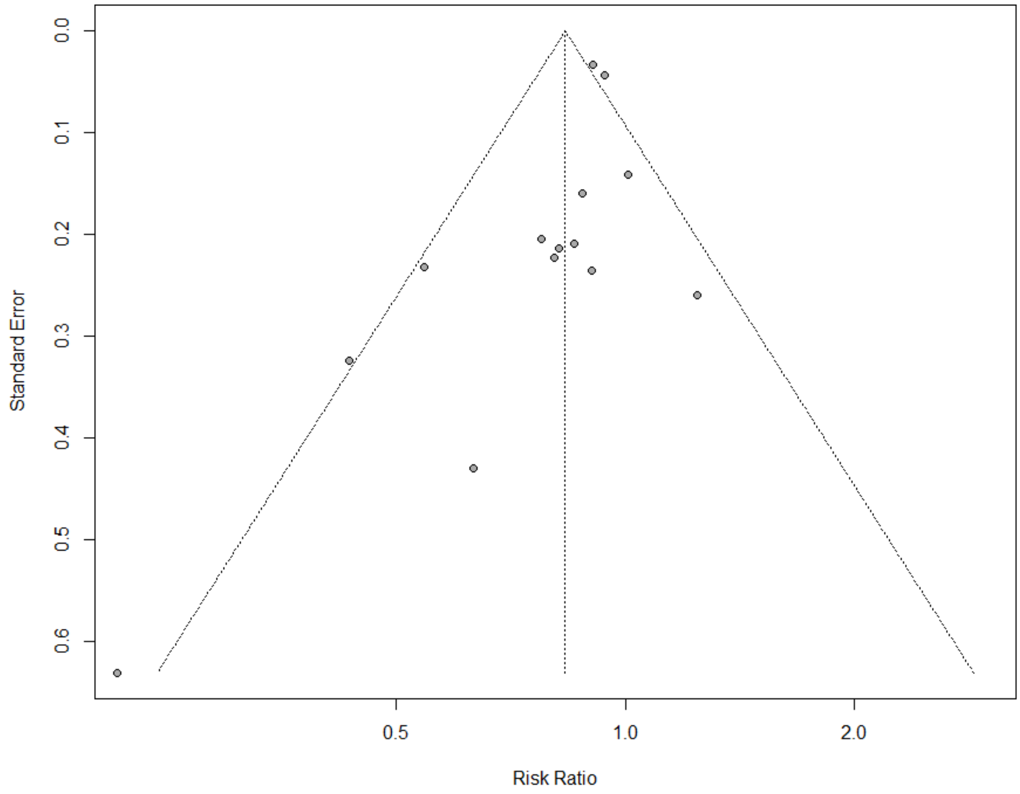

eFigure 29. **Funnel Plot for Thromboembolic Events**

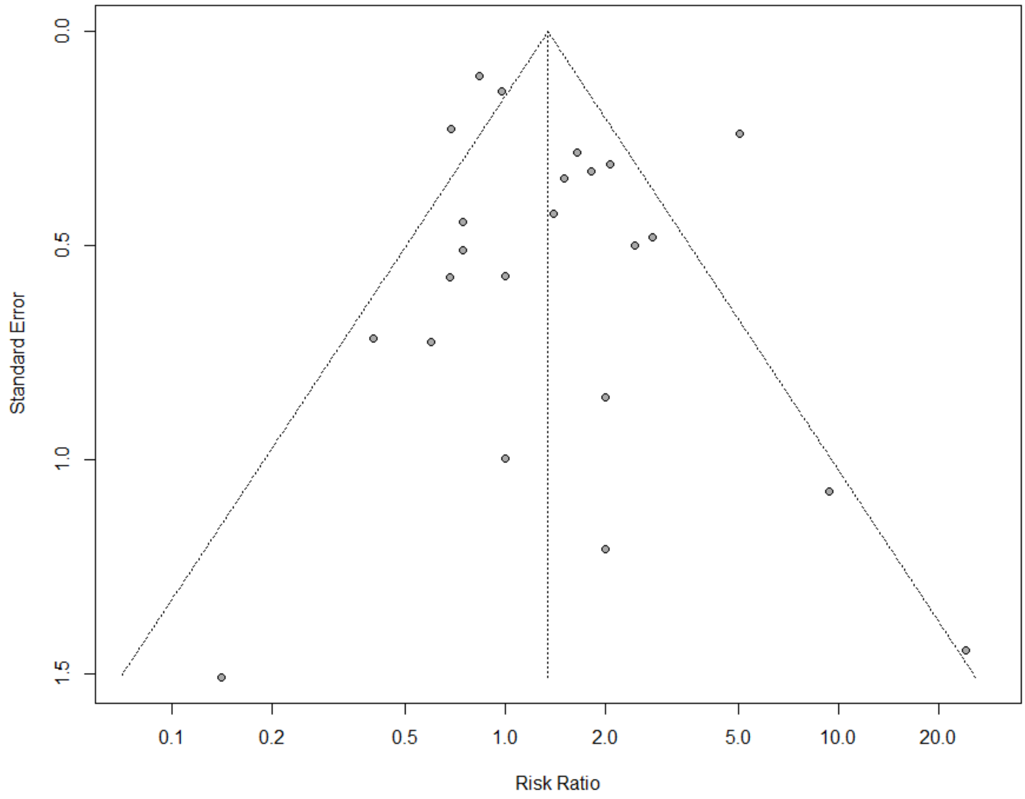

© 2022 Karl V et al. *JAMA Network Open*.

References

1. McGowan J, Sampson M, Salzwedel DM, Cogo E, Foerster V, Lefebvre C. PRESS Peer Review of Electronic Search Strategies: 2015 Guideline Statement. *J Clin Epidemiol*. Jul 2016;75:40-6. doi:10.1016/j.jclinepi.2016.01.021
2. Deeks JJ, Higgins JP, Altman DG, Group CSM. Analysing data and undertaking meta-analyses. *Cochrane handbook for systematic reviews of interventions*. 2019:241-284.
3. Langan D, Higgins JPT, Jackson D, et al. A comparison of heterogeneity variance estimators in simulated random-effects meta-analyses. *Res Synth Methods*. Mar 2019;10(1):83-98. doi:10.1002/jrsm.1316
4. Mathes T, Kuss O. A comparison of methods for meta-analysis of a small number of studies with binary outcomes. *Res Synth Methods*. Sep 2018;9(3):366-381. doi:10.1002/jrsm.1296
5. Kuss O. Statistical methods for meta-analyses including information from studies without any events-add nothing to nothing and succeed nevertheless. *Stat Med*. Mar 30 2015;34(7):1097-116. doi:10.1002/sim.6383
6. Balduzzi S, Rucker G, Schwarzer G. How to perform a meta-analysis with R: a practical tutorial. *Evid Based Ment Health*. Nov 2019;22(4):153-160. doi:10.1136/ebmental-2019-300117
7. Chakroun-Walha O, Samet A, Jerbi M, et al. Benefits of the tranexamic acid in head trauma with no extracranial bleeding: a prospective follow-up of 180 patients. *European Journal of Trauma & Emergency Surgery*. 2019;45(4):719-726.
8. CRASH-3 trial collaborators. Effects of tranexamic acid on death, disability, vascular occlusive events and other morbidities in patients with acute traumatic brain injury (CRASH-3): a randomised, placebo-controlled trial. *Lancet*. 2019;394(10210):1713-1723.
9. Rowell SE, Meier EN, McKnight B, et al. Effect of Out-of-Hospital Tranexamic Acid vs Placebo on 6-Month Functional Neurologic Outcomes in Patients With Moderate or Severe Traumatic Brain Injury. *JAMA*. Sep 8 2020;324(10):961-974. doi:10.1001/jama.2020.8958
10. Yutthakasemsunt S, Kittiwatanagul W, Piyavechvirat P, Thinkamrop B, Phuenpathom N, Lumbiganon P. Tranexamic acid for patients with traumatic brain injury: a randomized, double-blinded, placebo-controlled trial. *BMC Emergency Medicine*. 2013;13:20.
11. Chan DYC, Tsang ACO, Li LF, et al. Improving Survival with Tranexamic Acid in Cerebral Contusions or Traumatic Subarachnoid Hemorrhage: Univariate and Multivariate Analysis of Independent Factors Associated with Lower Mortality. *World Neurosurgery*. 2019;125:e665-e670.
12. Morte D, Lammers D, Bingham J, Kuckelman J, Eckert M, Martin M. Tranexamic acid administration following head trauma in a combat setting: Does tranexamic acid result in improved neurologic outcomes? *The Journal of Trauma and Acute Care Surgery*. 2019;87(1):125-129.
13. Walker PF, Bozzay JD, Johnston LR, Elster EA, Rodriguez CJ, Bradley MJ. Outcomes of tranexamic acid administration in military trauma patients with intracranial hemorrhage: a cohort study. *BMC Emergency Medicine*. 2020;20(1):39.
14. CRASH-2 trial collaborators. Effects of tranexamic acid on death, vascular occlusive events, and blood transfusion in trauma patients with significant haemorrhage (CRASH-2): a randomised, placebo-controlled trial. *Lancet*. 2010;376(9734):23-32.
15. Guyette FX, Brown JB, Zenati MS, et al. Tranexamic Acid During Prehospital Transport in Patients at Risk for Hemorrhage After Injury: A Double-blind, Placebo-Controlled, Randomized Clinical Trial. *JAMA Surgery*. 2020;05:05.
16. Bardes JM, Palmer A, Con J, Wilson A, Schaefer G. Antifibrinolytics in a rural trauma state: assessing the opportunities. *Trauma Surgery & Acute Care Open*. 2017;2(1):e000107.
17. Boutonnet M, Abback P, Le Sache F, et al. Tranexamic acid in severe trauma patients managed in a mature trauma care system. *The Journal of Trauma and Acute Care Surgery*. 2018;84(6S Suppl 1):S54-S62.
18. Cole E, Davenport R, Willett K, Brohi K. Tranexamic acid use in severely injured civilian patients and the effects on outcomes: a prospective cohort study. *Annals of Surgery*. 2015;261(2):390-4.
19. Dixon A, Emigh B, Spitz K, et al. Does tranexamic acid really work in an urban US level I trauma center? A single level 1 trauma center's experience. *American Journal of Surgery*. 2019;218(6):1110-1113.
20. El-Menyar A, Sathian B, Wahlen BM, et al. Prehospital administration of tranexamic acid in trauma patients: A 1:1 matched comparative study from a level 1 trauma center. *American Journal of Emergency Medicine*. 2020;38(2):266-271.
21. Glover TE, Sumpter JE, Ercole A, et al. Pulmonary embolism following complex trauma: UK MTC observational study. *Emergency Medicine Journal*. 2019;36(10):608-612.
22. Harvin JA, Peirce CA, Mims MM, et al. The impact of tranexamic acid on mortality in injured patients with hyperfibrinolysis. *The Journal of Trauma and Acute Care Surgery*. 2015;78(5):905-9; discussion 909-11.
23. Howard JT, Stockinger ZT, Cap AP, Bailey JA, Gross KR. Military use of tranexamic acid in combat trauma: Does it matter? *The Journal of Trauma and Acute Care Surgery*. 2017;83(4):579-588.

© 2022 Karl V et al. *JAMA Network Open*.

24. Johnston LR, Rodriguez CJ, Elster EA, Bradley MJ. Evaluation of Military Use of Tranexamic Acid and Associated Thromboembolic Events. *JAMA Surgery*. 2018;153(2):169-175.
25. Khan M, Jehan F, Bulger EM, et al. Severely injured trauma patients with admission hyperfibrinolysis: Is there a role of tranexamic acid? Findings from the PROPPR trial. *The Journal of Trauma and Acute Care Surgery*. 2018;85(5):851-857.
26. Lewis CJ, Li P, Stewart L, et al. Tranexamic acid in life-threatening military injury and the associated risk of infective complications. *British Journal of Surgery*. 2016;103(4):366-73.
27. Luehr E, Grone G, Pathak M, Austin C, Thompson S. Administration of tranexamic acid in trauma patients under stricter inclusion criteria increases the treatment window for stabilization from 24 to 48 hours-a retrospective review. *International Journal of Burns & Trauma*. 2017;7(6):115-119.
28. Moore HB, Moore EE, Huebner BR, et al. Tranexamic acid is associated with increased mortality in patients with physiological fibrinolysis. *Journal of Surgical Research*. 2017;220:438-443.
29. Morrison JJ, Dubose JJ, Rasmussen TE, Midwinter MJ. Military Application of Tranexamic Acid in Trauma Emergency Resuscitation (MATTERs) Study. *Archives of Surgery*. 2012;147(2):113-9.
30. Morrison JJ, Ross JD, Dubose JJ, Jansen JO, Midwinter MJ, Rasmussen TE. Association of cryoprecipitate and tranexamic acid with improved survival following wartime injury: findings from the MATTERs II Study. *JAMA Surgery*. 2013;148(3):218-25.
31. Myers SP, Kutcher ME, Rosengart MR, et al. Tranexamic acid administration is associated with an increased risk of posttraumatic venous thromboembolism. *J Trauma Acute Care Surg*. Jan 2019;86(1):20-27. doi:10.1097/TA.0000000000002061
32. Neeki MM, Dong F, Toy J, et al. Tranexamic Acid in Civilian Trauma Care in the California Prehospital Antifibrinolytic Therapy Study. *The Western Journal of Emergency Medicine*. 2018;19(6):977-986.
33. Neeki MM, Dong F, Toy J, et al. Safety and Efficacy of Hospital Utilization of Tranexamic Acid in Civilian Adult Trauma Resuscitation. *The Western Journal of Emergency Medicine*. 2020;21(2):217-225.
34. Shiraishi A, Kushimoto S, Otomo Y, et al. Effectiveness of early administration of tranexamic acid in patients with severe trauma. *British Journal of Surgery*. 2017;104(6):710-717.
35. Swendsen H, Galante J, Utter G, Bateni S, Scherer L, Schermer C. Tranexamic Acid use in Trauma: Effective but not Without Consequences. 2013;
36. Valle EJ, Allen CJ, Van Haren RM, et al. Do all trauma patients benefit from tranexamic acid? *The Journal of Trauma and Acute Care Surgery*. 2014;76(6):1373-8.
37. Wafaisade A, Lefering R, Bouillon B, et al. Prehospital administration of tranexamic acid in trauma patients. *Critical Care (London, England)*. 2016;20(1):143.
38. Spahn DR, Bouillon B, Cerny V, et al. The European guideline on management of major bleeding and coagulopathy following trauma: fifth edition. *Crit Care*. Mar 27 2019;23(1):98. doi:10.1186/s13054-019-2347-3

© 2022 Karl V et al. *JAMA Network Open*.
